# Molecular mediators of motion: RNA–RBP networks in exercise-induced osteoarthritis protection

**DOI:** 10.3389/fgene.2026.1788918

**Published:** 2026-03-12

**Authors:** Yupeng Yang, Xue Wang, Xuchang Zhou, Ying Li, Yinghao Shen, Zhujun Mao, Junjie Liu, Mi Zheng

**Affiliations:** 1 Graduate School, Harbin Sport University, Harbin, Heilongjiang, China; 2 Department of Rehabilitation Medicine, The First Affiliated Hospital of Xiamen University, School of Medicine, Xiamen University, Xiamen, China; 3 School of Basic Medical Sciences, China Three Gorges University, Yichang, China; 4 College of Science and Technology, China Three Gorges University, Yichang, China

**Keywords:** biomarkers, exercise, exosomes, molecular mechanisms, non-coding RNAs, osteoarthritis, precision therapy, RNA metabolic reprogramming

## Abstract

Osteoarthritis (OA) is a relatively common chronic degenerative disease of joints that was originally considered an imbalance between mechanical loads and tissue repair. Emerging evidence indicates that exercise confers protection not merely through mechanical loading, but also by acting as a systemic regulator of RNA metabolism. This modulation mainly happens by regulating RNA–RNA-binding protein interaction networks that can regulate joint homeostasis and delay the OA process. This article is a review of current understanding of how physical activity alters networks of RNA–RNA-binding proteins (RBPs) in different joint-related tissues, such as cartilage, synovium, skeletal muscle, and systemic circulation, and changes the metabolic and inflammatory pathways necessary for joint health. The article will examine molecular mechanisms by which exercise induces RNA metabolism reprogramming and protection from OA. It also studies the promising prospects of RNA–RBP networks in early detection of OA and targeting innovative treatment strategies. By combining what we know about RNA–RBP interaction with exercise physiology, this overview could clear the way to personalized exercise interventions and novel RNA-targeted therapies for OA.

## Graphical Abstract

**Figure:**
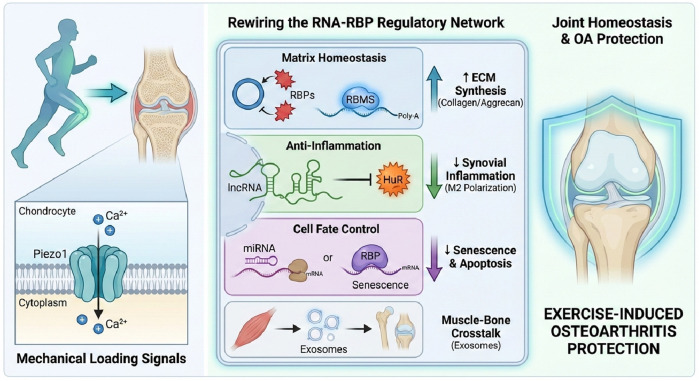
Exercise rewires the RNA–RBP interactome to protect against osteoarthritis. Mechanical loading signals (e.g., Piezo1) initiate a protective signaling cascade that remodels RNA–RBP networks across joint tissues. This systemic modulation orchestrates three key protective mechanisms: enhancing matrix anabolism, suppressing synovial inflammation, and inhibiting chondrocyte senescence, collectively restoring joint homeostasis.

## 1 Introduction

Osteoarthritis (OA) is a common joint degenerative disease that is a substantial cause of disability for millions of people. Both individuals and healthcare systems face a substantial disease burden (Zeng et al., 2025). Epidemiological data from global burden of disease studies show an increasing trend in the prevalence, incidence, and disability-adjusted life years of osteoarthritis during the past 3 decades, and it is projected to increase in the coming decades (Ding et al., 2024; Wang and Ye, 2024; Xie et al., 2025; Zhou J. et al., 2025). The burden of disease falls on older people and on areas with higher socio-demographic indices, driving the need for efficient prevention and control measures (Li Z. et al., 2025; Zeng et al., 2025). Women and middle-aged and older people are more prone to the disease, making the need for targeted interventions urgent (Ding et al., 2024; Sun et al., 2025). Although OA has a significant impact on quality of life and physical functioning, the current therapeutic strategies are mainly aimed at reducing symptoms, providing pain relief, and functional enhancement. However, these interventions are of low efficacy and incapable of inhibiting or improving the progression of the disease (Chen X. et al., 2024; Kim et al., 2025). The traditional pharmacologic agents, including NSAIDs, duloxetine, intra-articular injections, and so forth, have had little benefit in the long run and are accompanied by safety issues (Cao et al., 2020). Regenerative medicine methods such as mesenchymal stem cells and autologous conditioned serum have shown potential in preliminary clinical trials, but further research is needed to confirm (Chen X. et al., 2024; Jeyaraman et al., 2024). The development of disease-modifying osteoarthritis drugs remains an unmet need, as is research on targeting inflammation-driven, bone-driven, and cartilage-driven endotypes of OA (Makaram and Simpson, 2023; Oo, 2024; Rodriguez-Merchan, 2023). Non-pharmacological interventions, particularly exercise, have become a pillar of OA treatment. Exercise therapy is widely recommended in clinical guidelines for knee and hip OA and is helpful in reducing pain and improving physical function and quality of life (Lawford et al., 2024). Exercise is the only intervention that may have disease-modifying effects via its influence on joint tissue homeostasis and metabolic regulation (Deng et al., 2023; Du et al., 2024). The beneficial effects from exercise are not only from mechanical load but also from molecules and cells, by modulating inflammatory cytokines, and improving cartilage metabolism and regulation of subchondral bone remodeling (Du et al., 2024; Ma et al., 2024). Traditional Chinese exercises such as Tai Chi and Qigong can also help with the symptoms, tissue healing, and immune modulation, reducing joint inflammation (Du et al., 2024; Zhou et al., 2024). Even though the role of exercise is acknowledged, the exact molecular pathway by which exercise provides protection against joint disease has not been fully elucidated. A critical gap remains in understanding how macroscopic mechanical signals from exercise are translated into precise molecular outcomes. While traditional studies focus on signaling pathways, they often overlook the speed and complexity of cellular adaptation. This is where RNA–RBP networks become crucial. Unlike slow transcriptional changes, RBPs provide rapid “post-transcriptional” control, allowing joint cells (like chondrocytes) to instantly adjust their RNA stability and protein synthesis during the metabolic stress of exercise. Therefore, focusing on the RNA–RBP axis offers a unique and necessary rationale: it explains the “fast-response” mechanism that connects physical movement to long-term tissue repair. In the past, some studies found that RNA molecules, along with RBPs, also appear as dynamic regulators that respond to exercise produced by some mechanical or biochemical stimulations. RBPs serve as essential post-transcriptional regulators, orchestrating RNA splicing, stability, localization, and translation to ensure precise gene expression control (Flamand and Meyer, 2024; Kelaini et al., 2021). Exercise-induced modulation of RNA–mRNA localization, stability, and translation is a novel systemic reprogramming mode that may underlie the disease-modifying benefit of exercise in OA. This kind of all-round understanding is likely to find molecular targets for translation medicine, and on this basis, to develop new therapies that mimic or improve upon the protective effects of exercise in OA while maintaining joint integrity, reducing inflammation, and allowing healing (Wu et al., 2025). Therefore, this review hopes to integrate the current knowledge on the epidemiological burden of OA, the clinical efficiency of exercise as a disease-modifying intervention, and the molecular mediators involving the RNA–RBP network. We will study the molecular mechanism by which exercise regulates RNA metabolism to maintain joint tissue homeostasis and explore the translation prospects of this mechanism for OA treatment.

## 2 Basic RNA–RBP regulatory network in joint homeostasis

### 2.1 RNA diversity in joint tissues

There are many RNA species in joint tissues, and these species are responsible for maintaining joint homeostasis and mediating the pathogenesis of OA and RA. Among the RNAs, circular RNAs (circRNAs) have emerged as pivotal regulators to maintain the stability of the cartilage. Protective circRNAs help maintain the stability of the cartilage matrix by governing the synthesis of proteoglycans, which are critical components of the extracellular matrix (ECM) that give cartilage its load-bearing capacity. For example, some specific circRNAs have been found to regulate the expression of genes related to proteoglycan synthesis, so as to maintain the stability of the cartilage matrix and inhibit the degradation characteristics of OA (Ghanekar and Sadasivam, 2022). These circRNAs function as molecular “sponges” for miRNAs and act as scaffolds for RNA-binding proteins (RBPs) to modulate post-transcriptional modifications.

Another class of regulatory RNAs involved in joint inflammation and tissue remodeling is long non-coding RNAs (lncRNAs). Certain lncRNAs specifically modulate synovial inflammation by targeting inflammatory signaling pathways in fibroblast-like synoviocytes (FLSs), the main effector cells in synovitis. For instance, the lncRNA HAFML binds to the RBP HuR to stabilize mRNAs coding for proteins involved in FLS migration and invasion, both key aspects of joint destruction in rheumatoid arthritis (RA) (Xu et al., 2023). Furthermore, lncRNAs such as PVT1 work as competing endogenous RNAs (ceRNAs), whereby they sponge miRNAs like miR-140 and promote the expression of matrix-degrading enzymes (such as ADAMTS5 and MMP13) in chondrocytes to accelerate the degradation of the ECM in OA (Yao et al., 2022). These findings demonstrate that lncRNAs can participate in the organization and modulation of inflammatory reactions and matrix reconstruction processes in joint tissues.

MicroRNAs (miRNAs) are critical post-transcriptional regulators that determine chondrocyte fate, including survival, apoptosis, proliferation, and differentiation. Key miRNAs that regulate chondrocyte viability and balance the anabolic and catabolic activities of the cartilage have been identified. Dysregulation of these miRNAs results in enhanced chondrocyte apoptosis and impaired regeneration, which facilitates the progression of OA (Gu et al., 2023). The mutual influence among miRNAs, lncRNAs, circRNAs, and other RNA species is established in a complex regulatory network and jointly regulates gene expression.

Emerging research reveals that certain circRNAs and mRNAs can encode short functional peptides, thereby expanding the functional repertoire of non-coding RNAs. These peptides are increasingly recognized for their roles in modulating cellular metabolism and signaling pathways essential for joint homeostasis (Zhang C. et al., 2025)

Advances in single-cell RNA sequencing (scRNA-seq) and multi-omics technologies have significantly enhanced our understanding of cellular and molecular heterogeneity within joint tissues. These technologies enable the characterization of unique transcriptomic signatures across diverse cell types, detailing the expression patterns of various RNA species. For instance, scRNA-seq studies have identified several subsets of synovial macrophages and fibroblasts with distinct RNA expression signatures that are involved in inflammation and tissue remodeling in OA and RA (Laouteouet et al., 2025; Zhang et al., 2023). Moreover, integrative analysis between chromatin accessibility and transcriptomics has identified a regulatory network controlling joint cell RNA expression (Li H. et al., 2025).

Joint tissues harbor a diverse array of RNA species that collectively maintain joint integrity and promote OA pathogenesis. As summarized in Table 1, distinct RNA classes exert specialized regulatory functions: circRNAs modulate ECM metabolism by promoting synthesis or inhibiting degradation; lncRNAs regulate synovial pro-inflammatory responses and enhance target mRNA stability; and miRNAs govern the activity and polarization of chondrocytes and macrophages, while long non-coding RNAs may help modulate the synovium’s pro-inflammation response. Some can enhance the stability of target mRNA molecules and promote ECM formation; Finally, microRNAs have the functions of governing the activity and polarization of chondrocytes and macrophages, respectively, to achieve a moderate coexistence of the synthesizing and removing processes for the joint. There is some evidence that some circRNAs and mRNAs can code for functional peptides, which may further expand the scope of RNA regulation in joint biochemistry. These various types of RNAs interact with each other all the time and form networks that maintain the health of the joint and enable the RNA to respond to pathogenic stimuli like inflammation or mechanical stress (Kong et al., 2021).

**TABLE 1:** Key RNA types in joint tissues, their core regulatory functions, and corresponding references.

RNA type	Specific molecular example	Core function	Reference
Circular RNAs (circRNAs)	circPRKCH, circ-slain2, and hsa_circ_0000448	Regulate cartilage ECM synthesis and degradation, inhibit chondrocyte apoptosis, participate in inflammatory signaling pathways, and act as miRNA sponges	Hu et al. (2019); Pan et al. (2023); Que et al. (2022)
Long non-coding RNAs (lncRNAs)	HAFML, PVT1, WDR11-AS1, and MIR31HG	Regulate synovial inflammation, stabilize target mRNA expression, promote ECM synthesis, and inhibit AKT inflammatory signaling pathway	Cao L. et al. (2021); Huang et al. (2023); Xu et al. (2023); Yao et al. (2022)
MicroRNAs (miRNAs)	miR-140, miR-221-3p, miR-145, and miR-27a-3p	Regulate chondrocyte proliferation/apoptosis, modulate macrophage polarization, and promote bone anabolic metabolism	Que et al. (2022); Quero et al. (2019); Yao et al. (2022); Zhang B. et al. (2025)
circRNAs/mRNAs encoding peptides	Unknown (functional peptides)	Regulate chondrocyte metabolism, participate in cellular signaling pathways, and maintain joint tissue homeostasis	Zhang M. et al. (2025)

This table summarizes the main RNA species involved in joint homeostasis and osteoarthritis pathogenesis, including their representative molecular examples, critical regulatory roles in joint tissues such as cartilage and synovium, and relevant references. The regulatory functions listed are primarily derived from *in vitro* and *in vivo* experimental evidence in the cited studies.

To conclude, RNA diversity of joint tissue includes many protective circRNAs that guard cartilage ECM, inflammation-regulating lncRNAs in the synovium, miRNAs deciding the fate of chondrocytes, and coding circRNAs/mRNAs creating functional peptides. These different types of RNA interact with each other via RNA–RBP and epigenetic regulation, which regulates their joint homeostasis and pathologic processes in tissues. To comprehend such complex RNA nets is expected to provide direction for targeted, effective interventions to block or minimize joint diseases such as OA and RA.

### 2.2 Key RNA-binding proteins and their functions

RNA-binding proteins (RBPs) are central orchestrators of post-transcriptional gene regulation, governing essential processes such as alternative splicing, mRNA stability, subcellular localization, and translational initiation (Zigdon et al., 2024). In the context of osteoarthritis (OA), a degenerative joint disease characterized by progressive cartilage loss and chronic inflammation, RBPs serve as indispensable regulators of chondrocyte phenotypes and the maintenance of cartilage homeostasis. Notably, several specific RBPs have emerged as key mediators that modulate RNA metabolism, thereby profoundly influencing the pathophysiology and progression of OA.

### 2.3 Heterogeneous nuclear ribonucleoprotein family: regulation of RNA splicing, stability, and nucleocytoplasmic transport affecting chondrocyte stress response

The heterogeneous nuclear ribonucleoprotein (HNRNP) family is a group of RBPs that primarily participate in the processing of pre-mRNA, including pre-mRNA alternative splicing, mRNA stability, nuclear–cytoplasmic transport, etc. They bind to heterogeneous nuclear RNA and determine the functional fate and metabolic outcome of these RNAs. In OA, the members of the HNRNP family alter the response of chondrocytes to cell stress and inflammation, which are the main causes of cartilage damage. HNRNPD promotes chondrocyte senescence and OA progression through upregulating FOXM1 (Jiang et al., 2024), a transcription factor implicated in mitochondrial dysfunction and cellular aging. Similarly, HNRNPs may regulate the stability and translation of mRNAs encoding inflammatory mediator proteins and extracellular matrix proteins and thus modulate chondrocyte survival and maintain the integrity of the cartilage matrix (Wang H. et al., 2023; Zhang S. et al., 2024). Dynamic regulation of RNA splicing by HNRNPs may produce protein isoforms that either prevent or worsen cartilage injury. Moreover, HNRNPs are a part of stress granules and of RNA–protein complexes that are involved in the regulation of the cellular adaptation to oxidative and inflammatory stress, common in OA pathogenesis (Goswami et al., 2023). Therefore, the HNRNP family serves as a pivotal nexus between RNA metabolism and chondrocyte stress responses and influences the course and progression of OA.

### 2.4 RBMS proteins: involvement in mRNA stability and translational control linked to cartilage matrix protein expression

The RNA-binding motif, single-stranded interacting (RBMS) protein family, constitutes another critical group of regulators governing mRNA stability and translation. RBMS proteins bind to specific sequences or structures of target mRNAs to modulate their half-life and translational efficiency. This regulatory capacity is specifically targeted to the expression of cartilage extracellular matrix proteins, such as collagen and aggrecan, which are required for the formation and maintenance of cartilage. RBMS-mediated dysregulation of RBMS-mediated mRNA stability leads to dysregulated synthesis of ECM proteins, causing the cartilage degradation seen in OA. For example, RBMS proteins may stabilize the mRNAs encoding anabolic factors and destabilize the mRNAs encoding matrix-degrading enzymes, thereby achieving fine regulation of the balance between cartilage anabolism and catabolism. Although direct studies of RBMS proteins in OA are scarce, the known functions of these proteins in mRNA regulation and evidence from related tissues indicate that they are important for maintaining the homeostasis of cartilage by post-transcriptional regulation of matrix protein expression (Eraso et al., 2023; Yoon et al., 2022). Furthermore, RBMS proteins may interact with long non-coding RNAs (lncRNAs) and microRNAs, adding a layer of complexity to the post-transcriptional regulation of chondrocyte gene expression in OA.

### 2.5 IGF2BP family: modulation of mRNA localization and translation involved in cellular metabolism and inflammatory responses

Insulin-like growth factor 2 mRNA-binding proteins (IGF2BPs) are a group of RBPs that orchestrate mRNA localization, stability, and translation, thereby modulating cellular metabolism and inflammatory pathways (Wang et al., 2021). IGF2BPs bind to specific regions within the 5′ and 3′ untranslated regions of target mRNAs to regulate their localization, stability, and translation efficiency. In the setting of OA, the IGF2BP family of members was shown to regulate the transcription of metabolic genes in chondrocytes as well as inflammatory signaling genes. For instance, IGF2BP2 is associated with adipogenesis and metabolic regulation processes, which intersect with OA pathogenesis through systemic and local metabolic alterations (Zhang P. et al., 2022). In addition, dysregulated IGF2BP expression may also impact the stability and translation of mRNAs encoding cytokines and matrix metalloproteinases to alter the inflammatory environment and matrix remodeling in osteoarthritic cartilage. Studies have also shown that IGF2BPs are involved in RNA methylation pathways and are able to interact with circular RNAs, which have also been shown to be regulatory RNAs in OA (Shi and Zhao, 2025; Singh et al., 2025). The IGF2BP family all function as key post-transcriptional regulators, regarding mRNAs and metabolic and inflammatory processes that contribute to the progression of osteoarthritis.

To systematically categorize the molecular targets and specific regulatory effects of these key RBPs in OA, their distinct mechanisms are summarized in Table 2. In addition to the HNRNP, RBMS, and IGF2BP families, other important RBPs, including HNRNP, RBMS, PABPC1, PUM1, PUM2, SND1, and YTHDF3, interact with target mRNAs to directly modulate cartilage integrity, chondrocyte survival, and synovial inflammatory responses. This comprehensive overview highlights the functional complexity of individual RBPs and underscores the depth of the RBP regulatory network in OA.

**TABLE 2:** Key RNA-binding proteins (RBPs) in osteoarthritis: families, molecular targets, regulatory effects, and corresponding references.

RBP family/Protein	Specific members	Molecular targets (RNAs/mRNAs)	Regulatory effects in OA pathogenesis	Reference
HNRNP family	HNRNPD (AUF1)	FOXM1 mRNA	Promotes chondrocyte senescence by upregulating FOXM1; induces mitochondrial dysfunction and cellular aging; accelerates OA progression	Jiang et al. (2024)
	HNRNPA1	TRIM37 mRNA and TRAF6-related mRNAs	Stabilizes TRIM37 mRNA; mediates TRAF6 ubiquitination to alleviate cartilage inflammation and degradation	Deng H. et al. (2025)
	HNRNPQ	Autophagy-related mRNAs (ATG5 included)	Regulates autophagosome biogenesis; mutations impair autophagy, contributing to age-related joint tissue dysfunction	Ishtayeh et al. (2023)
RBMS proteins	RBMS1/2/3	Collagen, aggrecan, and matrix-degrading enzyme mRNAs	Stabilizes mRNAs of anabolic ECM proteins; destabilizes mRNAs of catabolic enzymes (MMPs/ADAMTS); balances cartilage synthesis and breakdown	Eraso et al. (2023) ; Yoon et al. (2022)
IGF2BP family	IGF2BP2	mRNAs of cytokines, MMPs, and adipogenesis-related genes	Modulates mRNA localization and translation; regulates chondrocyte metabolism, inflammatory signaling, and systemic metabolic cross talk in OA	Shi and Zhao (2025); Singh et al. (2025); Zhang P. et al. (2022)
Other key RBPs	PABPC1	SOX9 mRNA (regulated by lncRNA WDR11-AS1)	Directly binds and stabilizes SOX9 mRNA; increases ECM synthesis (collagen/aggrecan); counteracts OA-related cartilage degeneration	Huang et al. (2023)
	PUM1	TLR4 mRNA	Suppresses TLR4 mRNA translation; attenuates NF-κB signaling and cellular senescence; protects cartilage integrity	Lv G. et al. (2022) ; Yoon et al. (2022)
	PUM2	FOXO3 mRNA	Binds to 3′-UTR of FOXO3 mRNA; reduces FOXO3 expression; promotes IL-1β-induced chondrocyte apoptosis and ROS generation	Wang D. et al. (2024)
	SND1	HSPA5 mRNA	Destabilizes HSPA5 mRNA; decreases GPX4 expression; induces chondrocyte ferroptosis and cartilage damage	Lv M. et al. (2022)
	YTHDF3	LRRC17 mRNA (m^6^A-modified)	Stabilizes m^6^A-mediated LRRC17 mRNA; activates STAT1 signaling; increases ROS and mitochondrial dysfunction; accelerates chondrocyte senescence	Tang et al. (2025)

This table summarizes core RBPs involved in osteoarthritis pathogenesis, covering major RBPs, families, and critical individual members. It includes their direct RNA/mRNA, targets, specific regulatory effects on joint tissues such as cartilage and synovium, and relevant supporting references. The regulatory effects described are primarily based on experimental evidence from *in vitro* cell studies and *in vivo* animal models of osteoarthritis, reflecting the functional diversity of RBPs in modulating RNA metabolism and joint homeostasis.

### 2.6 Synergistic roles of RNA–RBP networks in joint homeostasis

RNA-binding proteins (RBPs) form complex regulatory networks with the target RNA and jointly maintain the homeostasis of the whole organism by precisely tuning gene expression at the post-transcriptional level. A central component of this regulation involves governing the balance between the synthesis and degradation of the cartilage extracellular matrix (ECM). RBPs selectively bind to target transcripts bearing the instruction codes for making the ECM, as well as for creating matrix-degrading enzymes, thereby dictating mRNA stability and translation efficiency to sustain this critical balance (Shao et al., 2020). For example, poly(A)-binding protein cytoplasmic 1 (PABPC1) binds to SOX9 mRNA and encodes a master transcription factor for ECM synthesis. This interaction is modulated by the long non-coding RNA (lncRNA) WDR11-AS1. This stabilization of SOX9 mRNA increases ECM anabolism, thereby inhibiting osteoarthritic cartilage erosion (Huang et al., 2023). In contrast, inflammatory signals increase RBPs, such as ALKBH5, and via m^6^A RNA demethylation, modulating the stability of RUNX2 mRNA. This transcription factor leads to the expression of matrix metalloproteinases and ADAMTS enzymes, which in turn break down ECM and accelerate osteoarthritis (OA) progression (Lei et al., 2024; Nie et al., 2025). Collectively, RNA–RBP complexes act as molecular rheostats, dynamically governing the anabolic and catabolic processes of cartilage to maintain ECM homeostasis.

Beyond their role in cartilage matrix regulation, RBP–RNA interaction networks are critically important in determining whether inflammation is initiated within the synovium. Dysfunctional chondrocyte-derived exosomes enriched in lncRNAs such as OANCT bind to RNA demethylases such as FTO, influencing the stability of mRNAs encoding PIK3R5 that activate the PI3K/AKT/mTOR pathway, thus promoting macrophage polarization to a pro-inflammatory M1 type and aggravating synovitis in OA (Lv et G. al., 2022; Zhang et al., 2020). Furthermore, RBPs such as HuR stabilize mRNAs encoding proteins involved in migration and invasion of the fibroblast-like synoviocytes, contributing to synovial hyperplasia and inflammation of rheumatoid arthritis, which may be related to the synovitis of osteoarthritis (Xu et al., 2023). It shows that RNA–RBP complexes can be fine-tuned under certain circumstances to play a role in synovial immune response and inflammatory thresholds. Although these mechanisms are indispensable for maintaining joint homeostasis, they may also drive pathological disease progression (Yu et al., 2025; Zhou Y. et al., 2025).

Aging and cellular senescence add another layer of complexity to joint homeostasis by altering RNA–RBP interactions that regulate the senescence-associated secretory phenotype. RBPs, such as the m^6^A reader YTHDF3, stabilize LRRC17 mRNA, which activates STAT1 signaling to induce chondrocyte senescence and increase ROS and mitochondrial dysfunction, causing a more severe osteoarthritis pathology (Tang et al., 2025). Similarly, RBPs such as PABPN1 and HNRNPQ, which regulate the expression of autophagy-related genes, also show dysfunctions due to mutations; these may result in reduced autophagosome biogenesis and cause age-related cellular dysfunction of joint tissue (Ishtayeh et al., 2023). The interplay between RBPs and non-coding RNAs in SASP regulation is an important aspect of the maintenance of joint tissue homeostasis in the course of aging and OA development.

RNA–RBP networks function synergistically to establish a complex regulatory environment in the joint tissue system, while balancing ECM synthesis and breakdown, regulating synovial inflammatory responses, and controlling cellular senescence and SASP. The disruption of these networks occurs in conditions such as OA, highlighting the possibility of therapeutic intervention by targeting the RNA–RBP interactions to reverse joint disequilibrium and halt progression of the disease (Cho et al., 2024; Guo et al., 2025; Sun et al., 2024; Suo et al., 2025). In the future, multiple RBPs can be profiled at the same time using more advanced techniques such as TRIBE-STAMP, and our understanding of the dynamic RNA–RBP interactions will also be increased in joint biology (Flamand and Meyer, 2024; Jin et al., 2024)

## 3 Exercise-induced multi-tissue RNA–RBP network reconstruction

### 3.1 Exercise-mediated regulation of RNA–RBP networks across tissues

As a non-invasive and effective intervention, exercise can halt osteoarthritis progression by modulating RNA–RBP networks across various joint tissues (Jia et al., 2023). This systemic regulation is not only the mechanical sensation in the local cartilage, but also the crosstalk of tissue of muscle and bone, the internal anti-inflammatory activity of synovium, and the far-reaching regulation through circulating factors. Table 3 summarizes the regulatory effects from five aspects: mechanical sensing-triggered local responses of cartilage, bidirectional interaction of the muscle–bone axis, anti-inflammatory regulation of synovium, system-wide intercellular communication via exosomes, and epitranscriptomic modulation through RNA methylation (Lai et al., 2025; Wang H. et al., 2024; Zhang K. et al., 2025). Each of these aspects can exist with specific molecular mediators, including mechanosensitive channels, exosomal RNAs, and RNA-modifying enzymes, that in whole rewire RNA–RBP interactions to re-establish joint homeostasis (Chen L. et al., 2024; Esmaeili et al., 2021; Gao et al., 2022; Shang et al., 2021; Steinecker-Frohnwieser et al., 2023).

**TABLE 3:** Exercise-induced RNA–RBP network regulation across multiple tissues: mechanisms, molecular mediators, and references.

Regulatory dimension	Target tissue	Core regulatory mechanisms	Key molecular mediators	Biological effects in OA protection	Reference
Local tissue response (mechanical sensing)	Cartilage	1. Piezo1 ion channel activation induces mechanosensitive circRNA expression 2. ceRNA (circRNA/lncRNA) sponges miRNAs to modulate RBP–mRNA interactions 3. Primary cilia-mediated signaling intersects with RNA–RBP networks	Piezo1, circRNAs (Piezo1-associated), lncRNAs, miRNAs, PABPC1, SOX9, MMP3/13, and ADAMTS4/5	- Enhances synthesis of proteoglycans and collagen - Inhibits chondrocyte apoptosis and ECM degradation - Regulates PI3K/AKT/NF-κB inflammatory pathways	Cheng et al. (2020); Ling et al. (2025); Zhou et Z. al. (2026)
Muscle–bone axis cross talk	Skeletal muscle + bone	1. Exercise-induced myokines trigger exosomal RNA secretion 2. RBP-mediated post-transcriptional regulation of osteocalcin and bone anabolic factors 3. Bidirectional exosomal miRNA exchange between muscle and bone	Exosomal miRNAs (miR-34a, miR-27a-3p, miR-486-5p, and miR-222-3p), IGF2BP2, osteocalcin, and STAT3	- Promotes bone anabolic responses and osteoblast differentiation - Enhances muscle regeneration - Alleviates osteosarcopenia-related joint instability	Wang B. et al. (2023); Wang et al. (2025); Zhang B. et al. (2025)
Synovial anti-inflammatory regulation	Synovium	1. Induction of anti-inflammatory ncRNAs to suppress pro-inflammatory signaling 2. Modulation of RNA methylation (m^6^A) on inflammatory mediator mRNAs 3. RBP-regulated cytokine production in synovial macrophages/FLSs	miRNAs (miR-221-3p), lncRNAs (MIR31HG), RBPs (HuR/ELAVL1), TGM2 (m^6^A-modified), JAK3/STAT3, and AKT	- Shifts macrophages from M1 to an anti-inflammatory phenotype - Inhibits FLS migration and inflammatory molecule production - Attenuates synovitis and synovial hyperplasia	Cao L. et al. (2021); Lin et al. (2022); Quero et al. (2019); Wood et al. (2024)
Systemic intercellular communication	Systemic circulation + joint cells	1. Exercise remodels exosomal RNA cargo (circRNAs/miRNAs) for long-distance transport 2. Exosomal RNAs target host cell RBPs to rewire RNA–RBP networks 3. ADAR-mediated RNA editing optimizes RNA–RBP binding specificity	Exosomes, circRNAs (hsa_circ_0000448), miRNAs, RBPs (PUM1/PUM2, and HSPB1), ADARs, FOXO3, and COL5A1	- Enhances chondrocyte survival and ECM homeostasis - Inhibits pro-inflammatory pathways (TNF-α and NF-κB) - Improves RNA metabolic reprogramming in joint cells	Fu et al. (2024); Hu et al. (2019); Su et al. (2024)
Epitranscriptomic regulation	Multiple tissues (cartilage/synovium)	1. Exercise modulates m^6^A methylation of circRNAs/mRNAs 2. m^6^A readers/writers (YTHDF1/2 and ALKBH5) regulate RBP–mRNA stability	m^6^A-modified circRNAs (hsa_circ_0007259), m^6^A enzymes (ALKBH5 and YTHDF1/2), RUNX2, and SETD7 mRNA	- Suppresses matrix-degrading enzymes (MMPs/ADAMTS) - Inhibits chondrocyte autophagy suppression and inflammation - Enhances RNA–RBP network specificity	Li L. et al. (2025); Luo et al. (2023); Nie et al. (2025)

This table summarizes the multi-dimensional regulatory roles of exercise in modulating RNA–RBP networks for osteoarthritis protection. It covers key regulatory dimensions, corresponding target tissues, core mechanisms, critical molecular mediators, biological effects, and relevant references. The included mechanisms integrate mechanical sensing, inter-tissue cross talk, inflammatory regulation, systemic communication, and epitranscriptomic modification, reflecting the systemic and coordinated characteristics of exercise-induced joint protection. All regulatory effects are supported by experimental evidence from *in vitro* cell studies, *in vivo* animal models, and partial clinical cohort data.

**FIGURE 1:**
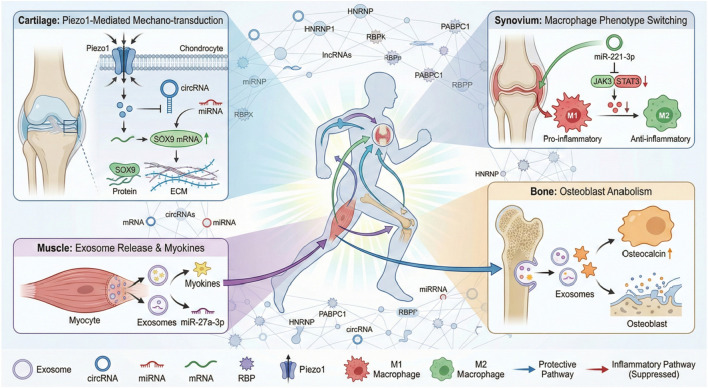
Schematic of the global RNA–RBP regulatory network in exercise-induced osteoarthritis protection. Mechanical loading stimulates mechanosensors (e.g., Piezo1) in cartilage to remodel RNA–RBP interactions, thereby promoting extracellular matrix (ECM) homeostasis. Simultaneously, the muscle–bone axis utilizes exosomal miRNAs to regulate bone remodeling. In the synovium, exercise-induced factors drive anti-inflammatory macrophage polarization. Systemic epitranscriptomic mechanisms (e.g., m^6^A methylation) further fine-tune these interactions network-wide. Note: The activation of these protective pathways is dependent on the magnitude and frequency of mechanical loading (dose–response) and may exhibit sex-specific variations, as detailed in the text. Abbreviations: OA, osteoarthritis; RBP, RNA-binding protein; ECM, extracellular matrix.

Importantly, these molecular pathways do not operate in isolation; rather, they function as an integrated network coupling local mechanical signals to distant biochemical responses to resist OA pathogenesis (Pérez-García et al., 2016; 2019; Li R. et al., 2022). As illustrated in Figure 1, we propose a global model in which exercise coordinately rewires RNA–RBP interactions across multiple tissues to restore joint homeostasis. The following sections will systematically elaborate on the core components of this framework: cartilage mechanotransduction, the muscle–bone axis, synovial immunomodulation, and systemic epitranscriptomic regulation.

### 3.2 Local responses of cartilage tissue

Articular cartilage has a special local response to mechanical stimuli that can help to maintain the joint’s self-balance as well as help in determining the path followed by the course of osteoarthritis (Shao et al., 2024; Yang et al., 2024; Zhang H. et al., 2022; Zhu et al., 2025). Many recent studies show and explain mechanical loading, which causes different expression changes in mechanosensitive circRNAs. We have also seen that the effect of mechanosensitive RNAs is important in regulating cartilage ECM (Chen et al., 2020; Hecht et al., 2019; Wang Z. et al., 2022; Wu et al., 2021; Xiang et al., 2020). Notably, the Piezo1 ion channel, as a mechanosensitive calcium channel, is now considered to be a mediator of this process (Marushack et al., 2025; Qin et al., 2024; Ren et al., 2023). The Piezo1 pathway is markedly activated in a rat model of knee OA caused by excessive mechanical loading, with the corresponding upregulation of Piezo1-associated circRNAs (Deng Z. et al., 2025; Wang S. et al., 2022; Yang et al., 2025). These circRNAs modulate the production of proteoglycans and collagen, which are key structural components of the cartilage ECM, thus determining the quality of cartilage repair (Zhou Z. et al., 2026). Piezo1 activation increases intracellular Ca^2+^ influx, and the downstream PI3K/AKT and NF-κB pathways are activated. The following pathways control transcription of genes involved with inflammation and matrix remodeling, and thus provide a mechanotransduction axis by which mechanical signals activate molecular responses in chondrocytes (Gan et al., 2024; Nims et al., 2024; Zhou Z. et al., 2026).

Competing endogenous RNAs have been considered to play an important role in regulating cartilage metabolism under mechanical stimuli by fine-tuning expression patterns of ceRNAs, such as circRNAs and lncRNAs, which function as molecular sponges that sequester miRNAs, and thus influence the availability of miRNAs to target mRNAs. This regulatory network is necessary for maintaining an anabolic–catabolic gene expression balance in cartilage. For example, circRNAs that are specific to circRNAs induced by mechanical stimuli can bind to RNA-binding proteins, which are important post-transcriptional regulatory factors (Pan et al., 2026). RBPs change the stability, splicing, and translation of mRNA, which then alters the expression of the mRNAs that create the ECM and those that destroy it. ceRNAs that are bound to RBPs interact with each other to create a regulating network that will regulate how chondrocytes will respond to different loads through adaptation and protection so that no cartilage deforms (Chen et al., 2017; Ling et al., 2025).

Mechanical stimulation influences transcription and regulation of apoptosis and ECM metabolism in chondrocytes through primary cilia. Primary cilia act as mechanosensors to convert a mechanical signal to a biochemical response. Studies using primary cilia-deficient mouse models showed that mechanical loading reduced cartilage degeneration and chondrocyte apoptosis via primary cilia-dependent signaling pathways that may involve RNA–RBP networks (Alvarez-Garcia et al., 2017; Hafsia et al., 2020; Ling et al., 2025). Mechanosensitive non-coding RNAs and RBPs might work with the primary cilia signal to direct cartilage homeostasis (Chaudhry et al., 2022; Crowe et al., 2016).

Exercise-induced mechanical stimulation modulates the expression of matrix metalloproteinases and aggrecanases, which are enzymes involved in ECM breakdown, through ceRNA- and RBP-mediated pathways (Cao Y. et al., 2021; Fioravanti et al., 2021; Shen et al., 2020). For instance, hydrogen-rich water treatment in OA models reduced expression of MMP3, MMP13, ADAMTS4, and ADAMTS5, while increasing expression of collagen type II and aggrecan. Antioxidants and anti-apoptotics appear to work through RNA regulatory networks affected by the mechanical environment (Cheng et al., 2020).

These findings emphasize the importance of mechanosensitive circRNAs and lncRNAs acting via ceRNA mechanisms and interaction with RBPs in regulating cartilage metabolism in response to mechanical loading (Bloks et al., 2024; Li et al., 2019). This complex RNA–RBP network regulates some important anabolic and catabolic genes and may be a factor in how moderate exercise protects against the development of OA. Understanding these molecular mediators can suggest target therapies that use mechanical signals to keep cartilage functioning and stop osteoarthritis (Shen et al., 2021).

Critical perspective: the dose–response paradox. While mechanical loading is a potent regulator of the RNA–RBP axis, it is crucial to distinguish between physiological loading (moderate exercise) and pathological overloading. Recent studies indicate that the same mechanosensitive channels, such as Piezo1, can drive divergent outcomes depending on the intensity of the stimulus. For instance, while moderate activation recruits protective networks to maintain homeostasis, excessive activation, such as that experienced during trauma or high-impact stress, may overwhelm these checkpoints, triggering HNRNP-mediated senescence pathways and Piezo1-associated inflammation (Wang S. et al., 2022). Therefore, the “protective” function of exercise is not intrinsic but conditional; it relies on an RNA–RBP threshold that differentiates adaptive remodeling from maladaptive degeneration (Bloks et al., 2024). Future research must delineate these specific molecular thresholds to optimize exercise prescriptions.

### 3.3 Skeletal muscle and “muscle–bone axis” RNA communication

Skeletal muscle and bone participate in a complex bidirectional communication network, often called the “muscle–bone axis,” that coordinates mechanical stimuli and biochemical signaling to maintain musculoskeletal homeostasis. Because of the increment of muscle movement caused by a workout, some skeletal muscle-originated factors called myokines are generated to affect bones and joints through many RNA methods. An emerging area within this communication is RNA modification, such as N^6^-methyladenosine (m^6^A) methylation, which dynamically influences RNA stability and translation. Physical exercise triggers the release of skeletal muscle exosomal miRNAs, which exert paracrine effects on nearby tissues and endocrine effects on remote muscles, bones, and cartilage. For example, muscle-derived exosomal miR-34a, miR-27a-3p, etc. facilitate muscle-to-bone communication, and bone-derived miR-486-5p promotes bone anabolism. Meanwhile, the muscle-derived exosomal miR-34a has a reciprocal effect on bone tissue (Zhang B. et al., 2025), showing the double-ended effect of RNA to bone–muscle communications. The exosomal miRNA exchange is regulated by physical activity, thus changing the miRNA profile to be on track to promote tissue regeneration and keep intact against osteosarcopenic degenerative states (Yu et al., 2024).

At the molecular level, RNA-binding proteins coordinate the expression of a sequence of protective proteins by determining the RNA stability, localization, and translation. RBPs adjust the lifespan of mRNAs that produce joint-protective substances, helping the body make adaptive responses to mechanical loading from exercise. For example, we modulate osteocalcin, an osteoblast-derived hormone that has properties for muscular regeneration through the muscle–bone signaling pathway. This might involve an RBP as well as post-transcriptional regulation (Wang B. et al., 2023). The restoration of osteocalcin in aged muscle by interventions such as ginkgolide B administration demonstrates that targeting RNA regulatory networks may have the therapeutic effect of improving muscle regeneration and thus joint health (Wang B. et al., 2023).

From the level of transcription, we can see that changes in muscle activity will also influence ECM remodeling and inflammatory signals. These processes also maintain the normal operation of cartilage and possibly delay the occurrence of OA. It was also proved that muscle-derived exosomal miRNAs, by regulating osteoblast differentiation and bone remodeling, were able to target transcription factors such as STAT3 with an anti-osteogenic role. This was demonstrated for miR-222-3p, which inhibits osteogenesis by targeting STAT3 (Wang et al., 2025). This demonstrates how RBPs and miRNAs coordinate to precisely regulate gene expression in musculoskeletal tissues and safeguard joints.

Apart from miRNA, long non-coding RNA and circular RNAs are competing endogenous RNAs that form competing endogenous RNA networks modulating myogenesis and possibly other muscle-related processes as well, indicating a wider range of RNA species in the regulation of muscle–bone communication (Li et al., 2024). These RNA molecules, which are regulated by RBPs, together influence muscle phenotype. It is possible that they could have an indirect effect on bone and cartilage homeostasis.

Exercise induces the release of a suite of RNAs contained within exosomes, which, under the aegis of RBPs that affect RNA stability and translation, bring about the creation of a protective molecular environment at distal joint sites via RBP-mediated regulation of RNA stability and translation. This RNA–RBP network promotes adaptive remodeling of bone and cartilage to prevent osteoarthritis. Understanding the molecular mediators can provide new possibilities for the development of RNA-targeted therapies that can utilize the muscle–bone axis to reduce the exercise-induced degeneration of joints and maintain the health of the musculoskeletal system (Hejazian et al., 2025).

### 3.4 Anti-inflammatory RNA regulation in the synovium

The synovium is a component of the joint structure that maintains joint homeostasis and regulates inflammation. It is also important in diseases such as osteoarthritis and rheumatoid arthritis. Exercise has been verified as interfering with synovial inflammation, partially by altering non-coding RNAs, such as microRNAs and long non-coding RNAs. These RNA species are important molecular mediators that tune inflammatory response precisely by targeting pro-inflammatory transcription factors and signaling pathways. For instance, anti-inflammatory miRNA such as miR-221-3p can be found in the synovium of rheumatoid arthritis patients, where they can regulate macrophage polarization through the inhibition of the JAK3/STAT3 signaling pathway and transforming the inflammatory macrophage type M1 into the anti-inflammatory macrophage type (Quero et al., 2019). Similarly, lncRNAs like MIR31HG are increased by anti-inflammatory treatment like tocilizumab, which acts by sponging miR-214 and therefore leading to a suppression of AKT signaling and the subsequent production of less inflammatory molecules from FLS (Cao L. et al., 2021). Exercise-induced mechanical stimuli may mimic or improve such regulatory networks to increase the expression of these anti-inflammatory ncRNAs, thereby inhibiting the activity of transcription factors such as NF-κB and STAT3 that promote synovial inflammation. In addition, the modulation of RNA methylation, such as N^6^-methyladenosine modification of mRNAs encoding inflammatory mediators such as TGM2, can also affect synovial cell proliferation and inflammatory signal transduction, suggesting that exercise may also help reduce the occurrence of synovitis (Lin et al., 2022; Xu et al., 2024).

**FIGURE 2:**
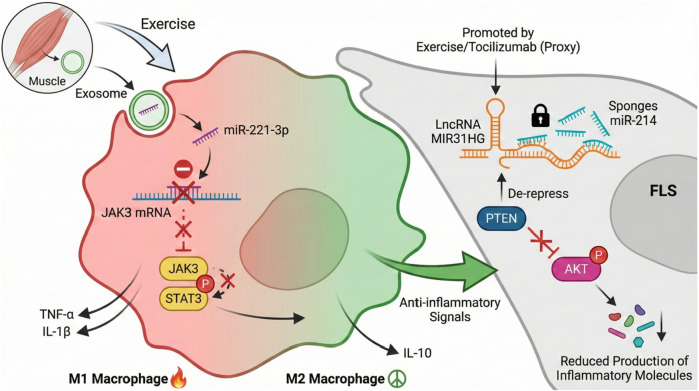
Exercise-induced RNA circuitry orchestrating anti-inflammatory signaling in the synovium. Exercise mitigates synovial inflammation by modulating specific RNA–RBP networks in key cell types. In synovial macrophages, exosomal miR-221-3p suppresses the JAK3/STAT3 pathway, promoting a shift from pro-inflammatory M1 to anti-inflammatory M2 polarization. In fibroblast-like synoviocytes (FLSs), the exercise-responsive lncRNA *MIR31HG* functions as a sponge for miR-214, thereby disinhibiting PTEN and suppressing AKT-driven inflammatory activation. These pathways are further regulated by RBPs (e.g., HuR) and m^6^A modifications. Abbreviations: FLS, fibroblast-like synoviocyte; PTEN, phosphatase and tensin homolog; m^6^A, N^6^-methyladenosine.

Molecular images can illustrate these processes as a circuit to protect the exercise synovium. In the synthesized pattern in Figure 2, the protection network is stabilized by the intracellular signaling network; exercise activates the circulated factor, including exosomal miR-221-3p and miR-221-3p suppresses JAK3/STAT3. In this case, M2 macrophages are formed. At the same time, exercise-upregulated lncRNAs such as MIR31HG sponge miR-214 in FLS cells, leading to PTEN-mediated AKT pathway inhibition and reduced inflammatory activation. RNA-binding proteins, including HuR, further stabilize key transcripts within this network (Li Z. et al., 2022), while m^6^A modifications add a layer of epitranscriptomic control (Lin et al., 2022; Xu et al., 2024). Collectively, this multi-level RNA–RBP network effectively increases the inflammatory threshold in the synovium, providing a molecular basis for the disease-modifying benefits of physical activity in OA.

### 3.5 Systemic and intercellular communication: indirect effects

Exercise-induced protection against osteoarthritis involves multiple systemic and intercellular communication pathways, which are mainly the circRNAs and miRNAs, and exosomes play a prominent role in this. Exercise changes how much RNA is deposited in these exosomes. Then, whether a cell in the joint does or does not take those exosomes up will also influence whether the joint is maintaining its cartilage homeostasis properly or if there is going to be joint degeneration and breakdown. circRNAs, a type of covalently closed circular RNA molecule, have been confirmed to be stable and abundant components in exosomes originating from joint tissues (Mao G. et al., 2021). For example, in temporomandibular joint osteoarthritis, specific circRNAs like hsa_circ_0000448 are upregulated and participate in inflammatory signaling pathways, especially the TNF-α pathway, through competing endogenous RNA mechanisms targeting relevant miRNAs. At the same time, circRNAs can also be bound by RNA-binding proteins, indicating that they may participate in post-transcriptional regulation after exosomal transfer (Hu et al., 2019). Similarly, miRNAs in exercise-induced exosomes can also regulate gene expression in chondrocytes by targeting mRNAs or binding RBPs, thereby regulating the cellular response to stress and inflammation. The joint cells take up these exosomes using endocytosis and receptor-mediated endocytosis, so the RNA cargo can affect intracellular signaling pathways to promote tissue repair and inhibit apoptosis.

Exosomal RNAs are partly chondroprotective by targeting host cell RBPs or mRNAs to modulate gene expression post-transcriptionally. RBPs are regulators of RNA metabolism and its regulators for RNA stability, as well as the spliceosome and translation. Several RBPs, such as PUM2 and PUM1, are implicated in both chondrocyte apoptosis and aging. PUM2 was upregulated in OA cartilage and in IL-1β-stimulated chondrocytes, which promoted apoptosis by binding to the 3′-UTR of FOXO3 mRNA to downregulate FOXO3 expression and promote ROS generation (Wang D. et al., 2024). On the other hand, PUM1 restrains TLR4 mRNA translation and weakens NF-κB signaling as well as cellular senescence, preserving cartilage structure (Yoon et al., 2022). Exosomal miRNAs and circRNAs can change the action of those RBPs, or they can simply go in and bind to a target mRNA, changing the RNA–RBP network in chondrocytes. For example, the RNA-binding protein HSPB1, which is low in expression in OA chondrocytes, binds to AU-rich elements of regulatory mRNAs on the extracellular matrix, such as COL5A1, and then regulates stability and translation (Fu et al., 2024). Exercise-derived exosomes could deliver RNA cargo that changes RBP expression or function, and this would enhance protective RNA–RBP networks that protect the ECM and stop the catabolic pathways in cartilage.

Exercise also affects RNA editing enzymes, such as adenosine deaminases acting on RNA that catalyze adenosine to inosine editing to change RNA secondary structure and function. ADAR-mediated editing can improve the specificity and stability of RNA–RBP interaction by modifying the RNA motif recognized by RBPs. Although there is not yet direct evidence that exercise increases ADAR activity in OA, the regulation of RNA editing provides a plausible basis for exercise fine-tuning an RNA–RBP network. RNA editing will influence the formation and function of circRNAs, miRNAs, etc., and their capacity to bind RBPs or target mRNAs, etc., and such dynamic regulation may improve the accuracy of post-transcriptional regulation in chondrocytes, thereby enhancing the ability of chondrocytes to resist inflammation and mechanical load. Given that aberrant RNA editing and RBPs are involved in OA pathogenesis (Su et al., 2024; Xiang et al., 2023), exercise-induced modulation of RNA editing enzymes could provide an important indirect means by which systemic and intracellular signaling imparts protection to articular cartilage.

To sum up, exercise-induced exosomes act as important mediators of exercise in the whole body and among cells through dynamically altering their circRNA and miRNA content for incorporation into joint cells and affecting RNA–RBP networks. The RNA molecule controls the survival of the chondrocyte, ECM homeostasis, and inflammatory response through RBPs or their mRNA substrates. Exercise could also regulate RNA editing enzymes such as ADARs to increase the diversity and specificity of RNA structural varieties, as well as RNA–RBP interaction specificity. It could also strengthen the post-transcriptional regulatory terrain that is able to defend cartilage against degeneration brought by osteoarthritis. The integrated RNA–RBP-exosome axis provides a potential target for OA therapy.

## 4 RNA–RBP network disruption in OA pathology

### 4.1 Dysregulation of protective RNA–RBP networks and activation of pathogenic axes

**FIGURE 3:**
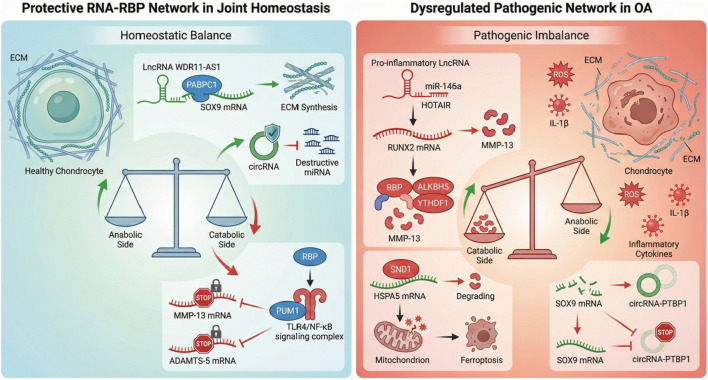
Dysregulation of RNA–RBP networks in osteoarthritis pathogenesis. The transition from health to disease involves a fundamental shift in RNA–RBP interactions. Left panel (homeostasis): Protective networks maintain joint integrity. For example, the lncRNA *WDR11-AS1* recruits PABPC1 to stabilize *SOX9* mRNA, promoting ECM synthesis, while PUM1 suppresses TLR4/NF-κB inflammatory signaling. Right panel (OA): Disrupted equilibrium drives pathogenesis. The loss of protective complexes (e.g., circRNA–PTBP1) and upregulation of pathogenic factors (e.g., *HOTAIR*, HNRNPD, and SND1) trigger chondrocyte senescence, ferroptosis, and matrix degradation. Abbreviations: ECM, extracellular matrix; OA, osteoarthritis; TLR4, Toll-like receptor 4.

The collapse of protective mechanisms and subsequent activation of pathogenic signaling axes represent the pivotal shift from joint homeostasis to osteoarthritis (OA) pathogenesis, as illustrated in Figure 3. For example, the circRNA–PTBP1 complex, which is important for post-transcriptional regulation in chondrocytes, is significantly decreased in quiescent or senescent cartilage. This loss breaks the stability of mRNAs with some important factors that create the ECM, making it difficult for the tissue to repair itself, leading to breakdown. A pathogenic landscape is also formed by the upregulation of pro-inflammatory, non-coding RNA, lncRNA HOTAIR, and miR-146a. These molecules make tough, pathological complexes with RBPs: HOTAIR works with PABPC1 to make pro-anabolic messages unstable, other disrupted RBPs like HNRNPD help chondrocytes age by making their transcripts of age-related proteins like FOXM1 persist (Jiang et al., 2024; Xiang et al., 2023). This double hit model, erosion of protection, and generation of pathogenesis form a self-perpetuating vicious cycle and promote the progression of OA. The following content will break down the molecular triggers of this imbalance in sequence, from the breakdown of cytoprotective networks to the explicit activation of detrimental RNA–RBP axes that orchestrate cartilage destruction and synovitis.

### 4.2 Dysregulation of protective RNA–RBP networks

In the context of osteoarthritis, the dysregulation of the protective RNA–RNA-binding protein network is a key factor that promotes cartilage degeneration due to joint inactivity or aging. A key example is that of the reduction in the expression of the circRNA–PTBP1 complex at these times, thus resulting in an imbalance in cartilage metabolism. circRNAs, as a kind of stable, covalently closed non-coding RNAs, have been gradually recognized as a new category of regulators that influence gene expression by competing for RBPs. PTBP1 is an RBP, which can affect the splicing and stability of RNA, and its pairing with circRNAs is an indispensable regulatory module to maintain the homeostasis of cartilage. The decrease of the circRNA–PTBP1 complex in quiescent or senescent RNA stability breaks this regulation balance, leading to a state where the genes involved in ECM synthesis and degradation cannot be post-transcriptionally controlled. This imbalance leads to the progressive loss of cartilage integrity of OA (Shen et al., 2023). In addition, the reduction of the RNA–RBP module also leads to a reduction in the repair capability of its intrinsic repairs of chondrocytes. RBPs take part in a substantial amount of RNA metabolism, including alternative splicing, mRNA stability, and translation. All of these need chondrocytes to be active and capable of responding to mechanical stimuli. The loss of function of these modules results in a decrease in the expression of anabolic factors and an increase in catabolic activity, thereby accelerating cartilage degeneration. Dysregulated RBPs like HNRNPA1 have been found to be involved in modulating the mRNAs that are crucial for inflammation and cell death pathways, thus causing the disease to progress. The downregulation of RBPs such as TIPARP in OA cartilage also shows a loss of protective post-transcriptional regulation. The cumulative effect of these changes is a failure to maintain the homeostasis of the cartilage, promoting the development of OA (Swahn et al., 2023). circRNAs are a sponge for microRNAs/RBPs. They form a complex regulatory microRNA–RBP that can modulate the expression of many target genes in chondrocytes. Breaking these networks, including the circRNA–miRNA–RBP axis, results in an abnormal gene expression profile, which favors inflammation, apoptosis, and ECM degradation. For example, circRNAs such as circTRIM25 and circTBX5 regulate chondrocyte ferroptosis and inflammatory responses through interactions with miRNAs and RBPs, thus highlighting the critical role of intact RNA–RBP networks in protecting cartilage. To sum up, the loss or dysfunction of the protective RNA–RBP network, such as the reduction of the circRNA–PTBP1 complex and the impairment of RBP activity, is at the root of metabolic imbalance and reduced repair capability of OA cartilage, especially under the state of inactivity or aging. Understanding of these molecular disruptions provides good opportunities for therapeutic intervention on the premise of restoring RNA–RBP balance for the purpose of protecting cartilage and inhibiting the progression of OA (Deng H. et al., 2025; Li et al., 2023; Swahn et al., 2023; Yi et al., 2022).

### 4.3 Activation of pathogenic RNA–RBP modules

In the process of osteoarthritis, a hallmark molecular event in the progression of OA is the upregulation of pro-inflammatory non-coding RNAs, particularly long non-coding RNAs such as HOTAIR, as well as microRNAs, including miR-146a. These RNAs also contribute to the formation of an abnormal RNA–RBP network that intensifies cartilage destruction and synovial inflammation. Pro-inflammatory lncRNAs such as HOTAIR are elevated, which have been shown to promote ECM breakdown by influencing gene expression post-transcriptionally, often by binding RBPs to regulate mRNA stability and translation. For example, HOTAIR links with RBPs like PABPC1, which binds to ECM-associated mRNAs such as SOX9 that act as a TF important for chondrocytic homeostasis. This interaction affects mRNA stability and ultimately protein expression, causing OA through defects in ECM synthesis and degradation (Huang et al., 2023). Similarly, miR-146a, which is known for its participation in inflammatory regulation, is upregulated in OA and modulates the expression of target genes involved in inflammatory signaling and cartilage catabolism. The binding of these miRNAs with RBPs increases their stability and functionality, resulting in a feed-forward loop that increases the inflammation and breakdown of the matrix.

At the same time, the expression and activity of some RBPs were also changed in OA, thereby reinforcing the pathogenic RNA–RBP modules. RBPs like HNRNPD and PUM2 have been shown to induce chondrocyte senescence via regulating the mRNA stability of FOXM1; they also induce a pro-inflammatory state by controlling the stability of mRNAs encoding TLR4. HNRNPD promotes the senescence of chondrocytes by increasing FOXM1 to accelerate the development of OA (Jiang et al., 2024). PUM2 restricts TLR4 mRNA so that NF-κB actions and inflammatory reactions are regulated. PUM2 represses TLR4 mRNA translation and controls NF-κB activity and inflammatory response; the downregulation of PUM2 within OA is responsible for increased inflammation and cartilage damage. The expression of RBPs is often dysregulated as a result of inflammatory stimuli like IL-1β, shifting the balance toward pro-inflammatory and catabolic states (Jiang et al., 2024).

RBPs such as SND1 also destabilize protective mRNAs like HSPA5, which are normally kept in check to prevent chondrocytes from suffering ferroptosis and oxidative stress. SND1-mediated degradation of HSPA5 mRNA results in decreased GPX4 expression, and the reduced expression of GPX4 leads to ferroptosis and contributes to the development of osteoarthritis (Lv M. et al., 2022). Pathogenic RNA–RBP interaction influences cell death pathways associated with OA.

Pro-inflammatory lncRNAs/miRNAs are more rapidly sequestered into the upregulated ribonucleoprotein complexes due to enhanced affinity for their respective RBPs, which leads to the formation of ribonucleoprotein complexes that promote inflammation and matrix catabolism. RNA-binding protein PUM2 binds to FOXO3 mRNA at 3'-UTR and inhibits translation, so IL-1β causes chondrocyte apoptosis, which is another event leading up to the formation of OA (Wang D. et al., 2024). Similarly, the RNA-binding protein DDX3X is upregulated in oxidative stress and promotes chondrocyte pyroptosis via activation of the NLRP3 inflammasome (Jiang et al., 2025).

Together, these findings indicate that OA progression is associated with the activation of pathogenic RNA–RBP modules manifested by the increased expression of pro-inflammatory lncRNAs, miRNAs, and their RBPs binding more frequently, which then form regulatory networks leading to cartilage destruction and synovial inflammation (Hu et al., 2024). These RNA–RBP interactions will modulate mRNA stability and translation. They also regulate the genes that code for the RNA-binding proteins involved in regulating inflammation, apoptosis, and ECM metabolism to control the molecular events of OA pathogenesis. Comprehending these complicated networks of RNA–RBPs offers possible therapeutic approaches to manipulate post-transcriptional gene regulation and alleviate the course of OA.

### 4.4 Metabolic dysregulation and inflammation feedback loop

Osteoarthritis (OA) is increasingly regarded as a multifactorial disease where metabolic dysregulation and chronic low-grade inflammation interweave in the joint microenvironment and make substantial changes to the homeostasis of all kinds of cells and gene expression regulation. Metabolic disorders, including poor glucose metabolism, mitochondrial damage, and an oxidative stress environment, are conducive to inflammatory reactions continuing. These metabolic disturbances break the fine balance of RNA post-transcriptional regulation, such as the interaction of RNA-binding proteins and the stability of RNA, which is important for maintaining the normal cellular function of joint tissues. For example, metabolic stress increases the level of ROS and changes the level of epigenetic modification, such as histone lactylation, which then affects transcriptional and post-transcriptional processes. Accumulated metabolites such as succinate and lactate function not only as metabolic intermediates but also as signaling molecules to regulate inflammatory gene expression and RNA processing. This dysregulation of metabolism damages the function of RBPs that control mRNA splicing, transport, and degradation, and thereby leads to a non-physiological expression of inflammatory mediators and matrix-degrading enzymes. As a result, the OA microenvironment has a compounded effect of metabolic stress, leading to an additional disturbance of RNA regulation and thus heightening joint tissue damage. The evidence from related chronic inflammatory and metabolic diseases shows that this kind of metabolic inflammation cross talk has some key signaling pathways (such as NF-κB and MAPK), as well as epigenetic regulators, that could change the RNA–RBP network, showing that post-transcriptional control under metabolic stress is complicated (Choi et al., 2025; Kojima et al., 2025; Zhou R. et al., 2026). Metabolic dysregulation, that is, inflammation interfering with RNA regulation, causes OA to be a disease.

**FIGURE 4:**
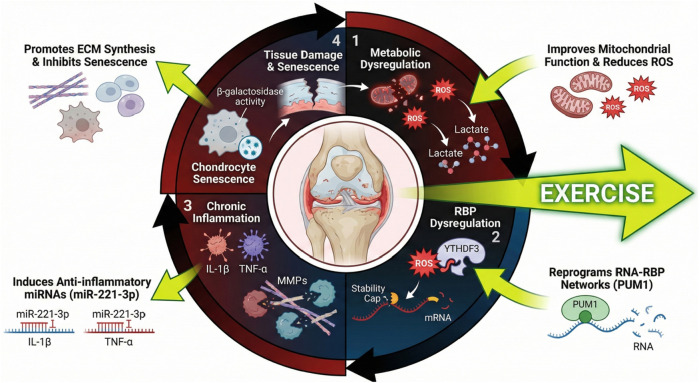
Targeting the metabolic–inflammatory–RBP cycle via exercise. A self-perpetuating feedback loop drives OA progression, where metabolic dysfunction amplifies pathogenic RNA–RBP interactions (e.g., *HOTAIR*, HNRNPD, and SND1) to fuel chronic inflammation. Exercise intervention: Physical activity disrupts this cycle by ameliorating metabolic stress, restoring protective complexes (e.g., circRNA–PTBP1), and suppressing inflammatory signaling to re-establish joint homeostasis. Abbreviations: OA, osteoarthritis; RBP, RNA-binding protein.

This synergistic impairment results in a self-perpetuating vicious cycle, visually summarized in Figure 4, which promotes the development of OA. The destruction of RNA–RBP interactions in the OA joint microenvironment establishes a cycle that accelerates disease progression. The role of RBPs within the cell is to dictate the fates of RNAs: where, when, and how often they are to be read (Xiang et al., 2023). This further shapes how the cell reacts to the external signals. Under conditions of metabolic dysregulation and inflammation, the expression and activity of RBPs are abnormally changed, resulting in maladaptive post-transcriptional regulation. For instance, oxidative stress, inflammatory cytokines, RBP expression, and function are all modulated, and in turn, they modulate the stability and translation of mRNAs that encode pro-inflammatory cytokines, matrix metalloproteinases, other catabolic factors, etc. (Xiang et al., 2023). That state of imbalance promotes the making of these inflammatory mediators, which causes even more metabolic stress and more cellular dysfunction. Moreover, some RBPs are also modified post-translationally by metabolic and inflammatory signals, thus changing their affinity and specificity for RNA (Yu et al., 2025; Zhou Y. et al., 2025). The feedback loop is a continual inflammatory status and metabolic disorder, causing the loss of cartilage, synovial inflammation, and subchondral bone remodeling characteristic of OA. Analogous mechanisms have been found in other chronic diseases with RNA–RBP network perturbations maintaining pathogenic inflammation and metabolic disruptions (Alzaid et al., 2025). Disrupting this RNA–RBP feedback loop could thus be a novel approach for breaking the loop of metabolic and inflammatory dysregulation in OA. Molecular mechanisms of RNA–RBP interactions in the framework of metabolic-inflammation cross talk must be clarified to develop countermeasures that would recover the equilibrium of the joint and stop the development of disease.

## 5 Translational medicine perspective: biomarkers and therapeutic targets of the RNA–RBP network

### 5.1 Development of novel biomarkers

**FIGURE 5:**
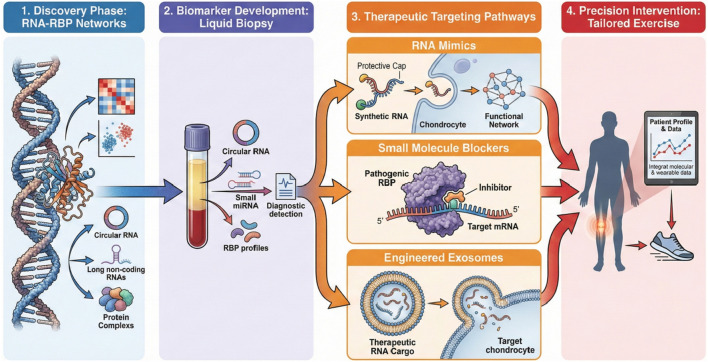
Translational roadmap of RNA–RBP networks in osteoarthritis precision medicine. The research pipeline progresses through four key stages: (1) discovery: multi-omics integration identifies disease-specific RNA–RBP signatures; (2) biomarker development: validation of circulating exosomal RNAs for early diagnosis and stratification; (3) therapeutic innovation: design of RNA mimics, small molecule inhibitors, and engineered exosomes; and (4) precision medicine: integration of individual molecular profiles with clinical data to guide personalized exercise prescriptions. Abbreviations: OA, osteoarthritis; RBP, RNA-binding protein.

Development of new biomarkers for the early diagnosis and disease stratification of osteoarthritis has begun to increasingly focus on molecular mediators responsive to mechanical stimulation, such as exercise. As seen in Figure 5, this effort is an important first step on the translation pipeline for RNA–RBP networks. Among the circulating and exosomal RNAs, circular RNAs have shown good prospects due to their stable property, tissue specificity, and regulatory function. circRNAs are produced through back-splicing events, resulting in covalently closed loop structures that are resistant to exonuclease degradation, thereby making circRNAs very stable in extracellular fluid. Their ability to act as microRNA sponges, interact with RNA-binding proteins, and influence transcriptional and epigenetic regulation positions them as key molecular players in OA pathogenesis and response to mechanical loading (Kour et al., 2022; Ren et al., 2020; Zhang Y. et al., 2022).

Exercise induces specific alterations in the expression profiles of circRNAs in chondrocytes and synovial tissues that can be detected in circulation and exosomes, thus providing a minimally invasive window into joint health. For instance, circZSWIM6 has been found to be upregulated in aging chondrocytes and in the development of OA. It regulates ECM metabolism and energy homeostasis through circZSWIM6–ribosomal protein S14 and AMPK signaling (Gong et al., 2023). Such circRNAs not only show pathological changes but also the good adaptations caused by physical exercise, and they can be used for the early detection of OA and disease phenotyping.

Epitranscriptomic modification of circRNAs, such as N^6^-methyladenosine methylation, also affects the stability and function of circRNAs. Differential m^6^A methylation patterns in circRNAs have been observed in inflammatory joint diseases, and hypermethylated circRNAs such as hsa_circ_0007259 are involved in rheumatoid arthritis pathogenesis, implying a possible parallel in OA (Luo et al., 2023). These modifications can be sensitive biomarkers for disease activity and therapeutic responses to exercise interventions.

In addition to circRNAs, RBPs and their PTMs represent a new class of biomarkers. RBPs control RNA splicing, stability, localization, and translation, and dysregulation of these factors promotes the progression of OA. RNA-binding protein GNL3, which can increase pro-inflammatory as well as angiogenic factors like IL-24 and PTN, will advance the progression of osteoarthritis (Zhu et al., 2021). Similarly, lncRNA WDR11-AS1 interacts with RBP PABPC1 to stabilize SOX9 mRNA and increase the synthesis of ECM to control the progression of OA (Huang et al., 2023). These results demonstrate the possibility of using profiles of RBP expression and modification states as indicators of the degree of OA and the response of the OA joint to mechanical stimuli.

Exercise modifies the expression of RBP, and the alternate splicing events of OA modulate apoptosis and cartilage homeostasis. Dysregulated RBPs, such as CUL4B and LAMA2, are also connected with apoptosis-related genes that are alternatively spliced (for example, XAF1 and BCL2L13), and these genes are important for OA (Zhang Z. et al., 2024). Monitoring the RBP modification spectra would then be able to provide prediction information on the success of exercise regimens to halt or reverse OA.

Furthermore, proteomic analysis of exosomes from synovial fluid has shown that there are differences in the expression of RBPs and other proteins in OA versus inflammatory arthritis, indicating that they may have diagnostic value (Huang et al., 2022). Due to the non-invasiveness of exosome collection and high throughput of molecular profiling, it is possible to observe changes in disease with exercise over time.

Together, the combination of circulating and exosomal circRNAs, their epitranscriptomic modifications, and the profiles of RBPs and their modifications form a potential biomarker platform to promote early OA diagnosis, subtyping the disease, and predicting an individual’s response to a certain exercise therapy. Future studies should aim to validate the molecular signatures in a large cohort and unify the methods of testing so that these results can be applied in the clinical setting (Mao X. et al., 2021).

### 5.2 Innovative therapeutic strategies

RNA drug design: mimicking exercise-induced protective circRNA/miRNA molecules to promote joint repair

Recent advances in the molecular understanding of osteoarthritis have highlighted the critical roles of circular RNAs (circRNAs) and microRNAs (miRNAs) in maintaining cartilage homeostasis. circRNAs are highly stable, tissue-specific non-coding RNAs formed by back splicing that function as miRNA sponges, RBP regulators, and transcriptional modulators. Their dysregulation is closely associated with OA onset, making them promising therapeutic targets (Kour et al., 2022). Exercise elicits protective molecular changes in joints, including the modulation of specific circRNAs and miRNAs that preserve cartilage integrity and exert anti-inflammatory effects. For instance, circPRKCH promotes ECM degradation via the miR-145–HGF axis; its knockdown alleviates OA progression by inhibiting chondrocyte apoptosis and matrix catabolism (Que et al., 2022). Similarly, circ-slain2 mitigates cartilage degradation and inflammation by regulating ER stress through ATF6, demonstrating anti-catabolic effects in OA models (Pan et al., 2023). These findings suggest that designing RNA therapeutics that mimic these exercise-induced protective profiles could restore ECM balance and chondrocyte survival. Synthetic circRNA or miRNA mimics could be engineered to function as molecular sponges or modifiers of pathogenic pathways, effectively replicating the molecular benefits of exercise. Such RNA therapeutics would leverage the inherent stability and tissue specificity of circRNAs to achieve targeted gene modulation in joint tissues, offering a novel avenue for OA treatment (Guo et al., 2025).

Targeting pathogenic RNA–RBP interfaces with small molecules or oligonucleotide drugs. RNA-binding proteins (RBPs) are pivotal post-transcriptional regulators governing mRNA stability, splicing, and translation, and their interactions are frequently dysregulated in OA. Aberrant RNA–RBP networks can drive inflammatory and catabolic cascades in chondrocytes. For example, the m^6^A demethylase ALKBH5 associates with the RBP YTHDF1 to stabilize *RUNX2* mRNA, a transcription factor that activates MMPs and ADAMTS enzymes, thereby accelerating ECM erosion. Furthermore, YTHDF2 stabilizes *SETD7* mRNA, enhancing IL-1β-induced chondrocyte injury via autophagy inhibition (Nie et al., 2025). These observations highlight the therapeutic potential of disrupting pathogenic RNA–RBP interactions. Strategies include designing small molecules or antisense oligonucleotides (ASOs) that specifically target the binding interfaces between pathogenic RNAs and RBPs. This approach prevents the stabilization and translation of deleterious mRNAs, thereby reducing inflammatory signaling. For instance, targeting the interaction between circPAFAH1B2 and the chaperone ClpB, which disrupts mitochondrial function, has been shown to restore mitochondrial homeostasis and chondrocyte survival (Bu et al., 2025). Similarly, blocking the binding of *SIRT1* antisense lncRNA to *SIRT1* mRNA can modulate cartilage-destructive enzymes (Tokura et al., 2025). These targeted approaches offer high specificity and the potential to fine-tune disease-driving molecular complexes.

Engineered exosome carriers: loading protective RNA cargo for joint-targeted delivery. Exosomes are nanoscale extracellular vesicles secreted by various cell types that facilitate intercellular communication by transporting proteins, lipids, and RNAs. They represent a novel, joint-specific delivery vehicle that mimics the molecular benefits of exercise. For example, exosomes delivering miR-145 have been shown to attenuate the pro-inflammatory effects of exogenous cold-inducible RNA-binding protein (CIRP) by inhibiting the TLR4/NF-κB/NLRP3 pathway (Sun et al., 2026), demonstrating their potential to modulate chondrocyte inflammation. Moreover, because exercise-induced myokines like irisin exert pro-chondrogenic and anti-apoptotic effects, exosomes engineered to carry RNAs that regulate irisin signaling could serve as “exercise mimetics” (Zhang M. et al., 2025). Engineered exosomes can be optimized for joint targeting, stability, and controlled release kinetics, overcoming the limitations of systemic RNA delivery. This strategy enables the local restoration of protective RNA networks within the joint microenvironment, promoting cartilage repair and ECM maintenance. Collectively, exosome-based RNA delivery systems hold great promise for translating the molecular mediators of exercise into precision OA therapies.

Current challenges and limitations Despite the therapeutic promise of targeting RNA–RBP networks, significant barriers remain for clinical translation. A primary challenge is the ubiquity of RBPs; proteins like HuR and IGF2BP family members are essential for normal physiology in multiple organs (Wang et al., 2021; Xu et al., 2023), implying that systemic inhibition could lead to severe off-target toxicity. Successful clinical application faces physical and biological hurdles. Intra-articular delivery is complicated by rapid clearance from the synovial space and the dense, avascular nature of the cartilage matrix, which severely limits the diffusion of therapeutic RNA cargo. Additionally, the potential immunogenicity of synthetic RNA vectors or engineered exosomes remains a safety concern that must be rigorously evaluated. Consequently, the development of advanced joint-specific delivery systems, such as cartilage-penetrating peptides or synovial-targeted engineered exosomes, is a prerequisite for safety and efficacy. Finally, a critical gap exists in the translational evidence base. Most current findings rely heavily on cross-sectional observations and rodent models, which do not fully recapitulate the biomechanical loading patterns and cartilage thickness of human joints. Longitudinal human clinical trials with specific RNA endpoints are notably scarce, limiting our ability to establish causality between exercise dose, RNA fluctuation, and clinical outcomes in patient populations (Espin-Garcia et al., 2022). The substantial heterogeneity in exercise protocols across existing studies that vary in frequency, intensity, duration, and loading type severely restricts direct cross-study comparisons. Consequently, future research must prioritize standardized reporting of exercise variables and rigorous validation in large-animal models (e.g., pigs or sheep) to bridge the chasm between molecular discovery and clinical application.

### 5.3 Future of personalized exercise prescription

The future direction of personalized exercise prescription in the management of osteoarthritis is to combine molecular-level information, that is, the individual-specific RNA–RBP (RNA-binding protein) network profile, and personalized exercise interventions. At present, the evidence for OA pathophysiology and patients’ response to exercise is abundant, and therefore, there are differences in the effects of standardized exercise program implementations. It has also recently been discovered that RNA–RBP networks are a key element in mediating the effects of exercise on joint protection, supporting progress in precision medicine. These networks regulate post-transcriptional gene expression and modulate cellular response to mechanical stimuli, inflammation, and tissue repair process, which is very important in the development of OA and recovery from the condition. By characterizing the unique expression and interaction patterns of the RNA–RBPs of an individual, clinicians may be able to infer what type of response the cartilage, synovium, or musculoskeletal tissue will have in an individual in response to certain types of exercise modalities, intensity, and duration.

Some recent studies have shown that exercise affects gene expression and epigenetics that can affect the OA process, but there is much variation among individuals due to genetic and molecular differences (Espin-Garcia et al., 2022). One example is exerkines, which are exercise-induced molecular signals, the production of which influences the inflammatory pathway and cartilage metabolism, and the potency of which is dependent on the exercise being done and the patient’s molecular milieu (Xu et al., 2025). Personalized exercise prescriptions guided by RNA–RBP network profiling can optimize the release of exerkines and the downstream protective effects, thereby maximizing benefits and reducing risks such as joint stress or aggravation of inflammation.

Technological advances, including wearable devices and mobile health apps that facilitate real-time monitoring and data collection of physical activity, provide the basis for adjusting exercise programs depending on patient feedback and molecular biomarkers (Gell et al., 2024; Hensley et al., 2023). Digital platforms could combine molecular information and physio/clinical molecules to make adaptable, patient-personalized physical activity interventions. However, there are still problems in converting RNA–RBP network data into operational clinical programs. More large-scale studies are required to confirm molecular signatures predictive of exercise response and develop algorithms robust enough to personalize exercise.

In addition, the incorporation of characteristics of RNA–RBP networks with other factors like muscle strength, biomechanics, and psychosocial factors will also be important to prescribe exercise holistically. For example, muscle weakness and altered gait biomechanics change joint loading and OA development, and their interaction with molecular profiles may guide personalized rehabilitation approaches (Peitola et al., 2025; Sacco et al., 2023). Psychological factors and patient–therapist relationships also mediate adherence and results (Lawford et al., 2021).

In the future, it will be possible to develop a personalized exercise prescription for OA based on the information of RNA–RBP networks to increase the therapeutic effect and safety of individualized interventions. This promises to improve on one-size-fits-all exercise recommendations by augmenting clinical work with molecular understanding. The potential will not be unleashed until there is multidisciplinary cooperation and the integration of molecular diagnostics and digital health technology with rigorous clinical validation to make personalized exercise the bedrock of OA management.

## 6 Conclusion and perspectives

We determined there was a relationship between exercise and RNA metabolism through the synergy and functions of the RNA-binding proteins that act as major molecular checkpoints and homeostasis control systems at the cellular level against osteoarthritis. This review has revealed that the systemic and multilayered modulation of RNA–RBP networks by physical activity is the basis not only for the protection function of exercise but also the transformation framework of the pathogenesis of OA. Experts consider that reconciling the various study outcomes, whether from molecular biology or clinical observation, can draw attention to the RNA–RBP interactions being pivotal for developing OA treatment biomarkers and therapeutic targets.

Progress in this field represents a paradigm shift. Previous research on OA mainly studied the biomechanics and inflammation pathomechanisms and did not address how RNA metabolism and RBPs function in tandem. This evidence, however, can be pieced together with known exercise benefits for joints to give a full picture of physical activity and its regulation of RNA metabolism for chondroprotection. The synthesis of the transcriptomics and proteomics study data, along with functional analysis, shows that exercise-induced reprogramming of RNA–RBP networks regulates the expression of genes, RNA stability, and protein translation in the joint tissues and thus reduces cartilage degeneration and inflammation.

This review notes that the variety of experiments and results must be reconciled. Some studies suggest exercise directly regulates the expression of some RBPs, and some studies put forward the idea of exercise exerting an effect on the activity of RBPs by exercising and circulating RNAs and other cellular communication. An integrative methodology that can integrate these three points of view and capture the dynamic *in vivo* RNA–RBP changes is needed. Finally, given the existence of OA phenotypic heterogeneity as well as patient differences in response to exercise interventions, stratified research designs that take into account the interplay between genetic, epigenetic, and environmental determinants of the RNA–RBP network are needed.

Integrating cutting-edge technologies will be pivotal. Single-cell multi-omics combined with long-read sequencing will allow for unprecedented resolution in tracing cell-type-specific RNA isoforms and RBP occupancy patterns under mechanical loading, defining the precise “molecular dose” of exercise. Moreover, to overcome the limitations of animal models, the adoption of human-derived organoids and “joint-on-a-chip” microphysiological systems offers a promising avenue for high-throughput screening. Crucially, translation to the clinic will require moving beyond a “one-size-fits-all” approach. Integrating wearable mechanosensors with AI-driven modeling could personalize exercise load and frequency, enabling “precision rehabilitation” that optimally engages protective RNA circuits in individual patients.

To sum up, the RNA–RBP network is a revolutionary thinking and operation network that can unite exercise physiology and OA pathobiology. By improving our knowledge of this mechanism, along with the new technologies, the possibilities of putting these discoveries into new and individualized prevention and treatment plans for osteoarthritis is an exciting avenue. This integrative strategy can not only expand scientific discussion, but also, in line with the goal of precise medicine, increase the patients’ survival and quality of life. Continuous research on exercise-regulated RNA–RBP reprogramming has moved to the front line of musculoskeletal system and medical care research.

## References

[B1] Alvarez-Garcia O. Matsuzaki T. Olmer M. Plate L. Kelly J. W. Lotz M. K. (2017). Regulated in development and DNA damage response 1 deficiency impairs autophagy and mitochondrial biogenesis in articular cartilage and increases the severity of experimental osteoarthritis. Arthritis Rheumatol. 69 (7), 1418–1428. 10.1002/art.40104 28334504 PMC5489357

[B2] Alzaid F. Arefanian H. Bahman F. Albeloushi S. Alhamar G. Mohammad A. (2025). Unraveling the RKIP-YY1 axis: immune crosstalk in the pathogenesis of metabolic disorders. Front. Immunol. 16, 1675699. 10.3389/fimmu.2025.1675699 41459495 PMC12739478

[B3] Bloks N. G. Harissa Z. Mazzini G. Adkar S. S. Dicks A. R. Hajmousa G. (2024). A damaging COL6A3 variant alters the MIR31HG-Regulated response of chondrocytes in neocartilage organoids to hyperphysiologic mechanical loading. Adv. Sci. (Weinh) 11 (36), e2400720. 10.1002/advs.202400720 39021299 PMC11423154

[B4] Bu Y. Zhao C. Qian Y. Chen L. Zhu K. Wu H. (2025). CircPAFAH1B2 induces chondrocytes mitochondrial dysfunction and promotes cartilage degeneration through binding molecular chaperone ClpB. J. Adv. Res. 75, 455–471. 10.1016/j.jare.2025.04.024 40286845 PMC12536665

[B5] Cao Z. Li Y. Wang W. Jie S. Hu X. Zhou J. (2020). Is lutikizumab, an Anti-Interleukin-1α/β Dual Variable domain immunoglobulin, efficacious for osteoarthritis? Results from a bayesian network meta-analysis. Biomed. Res. Int. 2020, 9013283. 10.1155/2020/9013283 33204726 PMC7661137

[B6] Cao L. Jiang H. Yang J. Mao J. Wei G. Meng X. (2021). LncRNA MIR31HG is induced by tocilizumab and ameliorates rheumatoid arthritis fibroblast-like synoviocyte-mediated inflammation *via* miR-214-PTEN-AKT signaling pathway. Aging (Albany NY) 13 (21), 24071–24085. 10.18632/aging.203644 34753831 PMC8610144

[B7] Cao Y. Tang S. Nie X. Zhou Z. Ruan G. Han W. (2021). Decreased miR-214-3p activates NF-κB pathway and aggravates osteoarthritis progression. EBioMedicine 65, 103283. 10.1016/j.ebiom.2021.103283 33714889 PMC7957119

[B8] Chaudhry N. Muhammad H. Seidl C. Downes D. Young D. A. Hao Y. (2022). Highly efficient CRISPR-Cas9-mediated editing identifies novel mechanosensitive microRNA-140 targets in primary human articular chondrocytes. Osteoarthr. Cartil. 30 (4), 596–604. 10.1016/j.joca.2022.01.005 35074547 PMC8987936

[B9] Chen W. K. Yu X. H. Yang W. Wang C. He W. S. Yan Y. G. (2017). lncRNAs: novel players in intervertebral disc degeneration and osteoarthritis. Cell. Prolif. 50 (1). e12313. 10.1111/cpr.12313 27859817 PMC6529103

[B10] Chen Y. Zhang L. Li E. Zhang G. Hou Y. Yuan W. (2020). Long-chain non-coding RNA HOTAIR promotes the progression of osteoarthritis *via* sponging miR-20b/PTEN axis. Life Sci. 253, 117685. 10.1016/j.lfs.2020.117685 32315726

[B11] Chen L. Liu J. Rao Z. (2024). FTO-Overexpressing extracellular vesicles from BM-MSCs reverse cellular senescence and aging to ameliorate osteoarthritis by modulating METTL3/YTHDF2-mediated RNA m6A modifications. Int. J. Biol. Macromol. 278 (Pt 1), 134600. 10.1016/j.ijbiomac.2024.134600 39122063

[B12] Chen X. Zheng J. Yin L. Li Y. Liu H. (2024). Transplantation of three mesenchymal stem cells for knee osteoarthritis, which cell and type are more beneficial? A systematic review and network meta-analysis. J. Orthop. Surg. Res. 19 (1), 366. 10.1186/s13018-024-04846-1 38902778 PMC11188250

[B13] Cheng S. Peng L. Xu B. Chen W. Chen Y. Gu Y. (2020). Protective effects of hydrogen-rich water against cartilage damage in a rat model of osteoarthritis by inhibiting oxidative stress, matrix catabolism, and apoptosis. Med. Sci. Monit. 26, e920211. 10.12659/msm.920211 31927559 PMC6977642

[B14] Cho Y. Kim H. Yook G. Yong S. Kim S. Lee N. (2024). Predisposal of interferon regulatory factor 1 deficiency to accumulate DNA damage and promote osteoarthritis development in cartilage. Arthritis Rheumatol. 76 (6), 882–893. 10.1002/art.42815 38268484

[B15] Choi W. Woo G. H. Kwon T. H. Jeon J. H. (2025). Obesity-driven metabolic disorders: the interplay of inflammation and mitochondrial dysfunction. Int. J. Mol. Sci. 26 (19), 9715. 10.3390/ijms26199715 41096980 PMC12525337

[B16] Crowe N. Swingler T. E. Le L. T. Barter M. J. Wheeler G. Pais H. (2016). Detecting new microRNAs in human osteoarthritic chondrocytes identifies miR-3085 as a human, chondrocyte-selective, microRNA. Osteoarthr. Cartil. 24 (3), 534–543. 10.1016/j.joca.2015.10.002 26497608 PMC4769094

[B17] Deng X. Xu H. Hao X. Liu J. Shang X. Xu T. (2023). Effect of moderate exercise on osteoarthritis. EFORT Open Rev. 8 (3), 148–161. 10.1530/eor-22-0119 36916731 PMC10026061

[B18] Deng H. Liu W. Xiang L. Wu J. (2025). HNRNPA1 promotes TRIM37 mRNA stability and mediates TRAF6 ubiquitination to alleviate osteoarthritis. Int. Immunopharmacol. 166, 115568. 10.1016/j.intimp.2025.115568 41014769

[B19] Deng Z. Li C. Hu S. Zhong Y. Li W. Lin Z. (2025). sdRNA-D43 derived from small nucleolar RNA snoRD43 improves chondrocyte senescence and osteoarthritis progression by negatively regulating PINK1/Parkin-mediated mitophagy pathway *via* dual-targeting NRF1 and WIPI2. Cell. Commun. Signal 23 (1), 77. 10.1186/s12964-024-01975-2 39934774 PMC11817878

[B20] Ding Y. Liu X. Chen C. Yin C. Sun X. (2024). Global, regional, and national trends in osteoarthritis disability-adjusted life years (DALYs) from 1990 to 2019: a comprehensive analysis of the global burden of disease study. Public Health 226, 261–272. 10.1016/j.puhe.2023.10.030 38134839

[B21] Du X. Fan R. Kong J. (2024). What improvements do general exercise training and traditional Chinese exercises have on knee osteoarthritis? A narrative review based on biological mechanisms and clinical efficacy. Front. Med. (Lausanne) 11, 1395375. 10.3389/fmed.2024.1395375 38841568 PMC11150680

[B22] Eraso P. Mazón M. J. Jiménez V. Pizarro-García P. Cuevas E. P. Majuelos-Melguizo J. (2023). New functions of intracellular LOXL2: Modulation of RNA-binding proteins. Molecules 28 (11), 4433. 10.3390/molecules28114433 37298909 PMC10254187

[B23] Esmaeili A. Hosseini S. Baghaban Eslaminejad M. (2021). Engineered-extracellular vesicles as an optimistic tool for microRNA delivery for osteoarthritis treatment. Cell. Mol. Life Sci. 78 (1), 79–91. 10.1007/s00018-020-03585-w 32601714 PMC11072722

[B24] Espin-Garcia O. Baghel M. Brar N. Whittaker J. L. Ali S. A. (2022). Can genetics guide exercise prescriptions in osteoarthritis? Front. Rehabil. Sci. 3, 930421. 10.3389/fresc.2022.930421 36188938 PMC9397982

[B25] Fioravanti A. Tenti S. Cheleschi S. (2021). MiR-214-3p, a novel possible therapeutic target for the pathogenesis of osteoarthritis. EBioMedicine 66, 103300. 10.1016/j.ebiom.2021.103300 33774326 PMC8024903

[B26] Flamand M. N. Meyer K. D. (2024). Simultaneous profiling of the RNA targets of two RNA-Binding proteins using TRIBE-STAMP. Methods Enzymol. 705, 127–157. 10.1016/bs.mie.2024.07.008 39389662

[B27] Fu Q. Li Y. Shi C. (2024). HSPB1 as an RNA-Binding protein mediates the pathological process of osteoarthritis. J. Orthop. Surg. Res. 19 (1), 156. 10.1186/s13018-024-04580-8 38429742 PMC10908047

[B28] Gan D. Tao C. Jin X. Wu X. Yan Q. Zhong Y. (2024). Piezo1 activation accelerates osteoarthritis progression and the targeted therapy effect of artemisinin. J. Adv. Res. 62, 105–117. 10.1016/j.jare.2023.09.040 37758057 PMC11331168

[B29] Gao W. Hasan H. Anderson D. E. Lee W. (2022). The role of mechanically-activated ion channels Piezo1, Piezo2, and TRPV4 in chondrocyte mechanotransduction and mechano-therapeutics for osteoarthritis. Front. Cell. Dev. Biol. 10, 885224. 10.3389/fcell.2022.885224 35602590 PMC9114637

[B30] Gell N. M. Smith P. A. Wingood M. (2024). Physical therapist and patient perspectives on Mobile technology to support home exercise prescription for people with arthritis: a qualitative study. Cureus 16 (3), e55899. 10.7759/cureus.55899 38601402 PMC11006223

[B31] Ghanekar Y. Sadasivam S. (2022). RNA editing-associated post-transcriptional gene regulation in rheumatoid arthritis. Bioinform Biol. Insights 16, 11779322221088725. 10.1177/11779322221088725 35462874 PMC9021465

[B32] Gong Z. Wang K. Chen J. Zhu J. Feng Z. Song C. (2023). CircZSWIM6 mediates dysregulation of ECM and energy homeostasis in ageing chondrocytes through RPS14 post-translational modification. Clin. Transl. Med. 13 (1), e1158. 10.1002/ctm2.1158 36604982 PMC9816529

[B33] Goswami B. Nag S. Ray P. S. (2023). Fates and functions of RNA-Binding proteins under stress. Wiley Interdiscip. Rev. RNA e1825, e1825. 10.1002/wrna.1825 38014833

[B34] Gu Y. Hu Y. Zhang H. Wang S. Xu K. Su J. (2023). Single-cell RNA sequencing in osteoarthritis. Cell. Prolif. 56 (12), e13517. 10.1111/cpr.13517 37317049 PMC10693192

[B35] Guo S. K. Suo J. Huang Y. Yin X. Wang J. Li L. (2025). Therapeutic circRNA aptamer alleviates PKR-Associated osteoarthritis. Sci. Bull. (Beijing) 70 (14), 2232–2236. 10.1016/j.scib.2025.02.027 40021383

[B36] Hafsia N. Forien M. Renaudin F. Delacour D. Reboul P. Van Lent P. (2020). Galectin 3 deficiency alters chondrocyte primary cilium formation and exacerbates cartilage destruction *via* mitochondrial apoptosis. Int. J. Mol. Sci. 21 (4). 1486. 10.3390/ijms21041486 32098291 PMC7073077

[B37] Hecht N. Johnstone B. Angele P. Walker T. Richter W. (2019). Mechanosensitive MiRs regulated by anabolic and catabolic loading of human cartilage. Osteoarthr. Cartil. 27 (8), 1208–1218. 10.1016/j.joca.2019.04.010 31009748

[B38] Hejazian S. S. Hejazian S. M. Mostafavi Montazeri S. S. Abediazar S. Zununi Vahed S. Barzegari A. (2025). Mesenchymal stem cell therapy in osteoarthritis and rheumatoid arthritis: a systematic review of exosomal microRNAs. Biologics 19, 747–785. 10.2147/btt.S571417 41509067 PMC12777084

[B39] Hensley C. P. Witte M. M. Cai J. Gruenke A. Pecze J. Mangefrida A. (2023). Assessment of Mobile health applications for management of knee and/or hip osteoarthritis using the Mobile application rating scale. J. Clin. Rheumatol. 29 (5), 245–253. 10.1097/rhu.0000000000001896 36256541

[B40] Hu Y. Zhu H. Bu L. He D. (2019). Expression profile of circular RNA s in TMJ osteoarthritis synovial tissues and potential functions of hsa_circ_0000448 with specific back-spliced junction. Am. J. Transl. Res. 11 (9), 5357–5374. 31632516 PMC6789216

[B41] Hu K. Wen H. Song T. Che Z. Song Y. Song M. (2024). Deciphering the role of LncRNAs in osteoarthritis: inflammatory pathways unveiled. J. Inflamm. Res. 17, 6563–6581. 10.2147/jir.S489682 39318993 PMC11421445

[B42] Huang Y. Liu Y. Huang Q. Sun S. Ji Z. Huang L. (2022). TMT-based quantitative proteomics analysis of synovial fluid-derived exosomes in inflammatory arthritis. Front. Immunol. 13, 800902. 10.3389/fimmu.2022.800902 35359923 PMC8961740

[B43] Huang H. Yan J. Lan X. Guo Y. Sun M. Zhao Y. (2023). LncRNA WDR11-AS1 promotes extracellular matrix synthesis in osteoarthritis by directly interacting with RNA-binding protein PABPC1 to stabilize SOX9 expression. Int. J. Mol. Sci. 24 (1), 817. 10.3390/ijms24010817 36614257 PMC9820994

[B44] Ishtayeh H. Galves M. Barnatan T. T. Berdichevsky Y. Amer-Sarsour F. Pasmanik-Chor M. (2023). Oculopharyngeal muscular dystrophy mutations link the RNA-Binding protein HNRNPQ to autophagosome biogenesis. Aging Cell. 22 (10), e13949. 10.1111/acel.13949 37559347 PMC10577562

[B45] Jeyaraman N. Jeyaraman M. Ramasubramanian S. Yadav S. Balaji S. Patro B. P. (2024). Autologous conditioned serum in knee osteoarthritis: a systematic review of current clinical evidence. Cureus 16 (9), e68963. 10.7759/cureus.68963 39385904 PMC11461807

[B46] Jia S. Yu Z. Bai L. (2023). Exerkines and osteoarthritis. Front. Physiol. 14, 1302769. 10.3389/fphys.2023.1302769 38107476 PMC10722202

[B47] Jiang H. Zhang Y. Hu G. Ji P. Ming J. Li Y. (2024). RNA-Binding protein HNRNPD promotes chondrocyte senescence and osteoarthritis progression through upregulating FOXM1. Commun. Biol. 7 (1), 1695. 10.1038/s42003-024-07407-8 39719453 PMC11668876

[B48] Jiang S. Yuan F. Zhou H. (2025). DDX3X activates chondrocyte pyroptosis to promote osteoarthritis progression. Cell. Biochem. Biophys. 83 (2), 1955–1962. 10.1007/s12013-024-01605-1 39592517

[B49] Jin H. Li C. Jia Y. Qi Y. Piao W. (2024). Revealing the hidden RBP-RNA interactions with RNA modification enzyme-based strategies. Wiley Interdiscip. Rev. RNA 15 (3), e1863. 10.1002/wrna.1863 39392204 PMC11469752

[B50] Kelaini S. Chan C. Cornelius V. A. Margariti A. (2021). RNA-binding proteins hold key roles in function, dysfunction, and disease. Biol. (Basel) 10 (5), 366. 10.3390/biology10050366 33923168 PMC8146904

[B51] Kim E. H. Jeon S. Park J. Ryu J. H. Mobasheri A. Matta C. (2025). Progressing future osteoarthritis treatment toward precision medicine: integrating regenerative medicine, gene therapy and circadian biology. Exp. Mol. Med. 57 (6), 1133–1142. 10.1038/s12276-025-01481-6 40588525 PMC12229678

[B52] Kojima R. Hirata Y. Ashida R. Takahashi M. Matsui R. Hama K. (2025). Elaidic acid drives cellular senescence and inflammation *via* lipid raft-mediated IL-1R signaling. iScience 28 (9), 113305. 10.1016/j.isci.2025.113305 40894877 PMC12396248

[B53] Kong H. Sun M. L. Zhang X. A. Wang X. Q. (2021). Crosstalk among circRNA/lncRNA, miRNA, and mRNA in osteoarthritis. Front. Cell. Dev. Biol. 9, 774370. 10.3389/fcell.2021.774370 34977024 PMC8714905

[B54] Kour B. Gupta S. Singh R. Sophiarani Y. Paul P. (2022). Interplay between circular RNA, microRNA, and human diseases. Mol. Genet. Genomics 297 (2), 277–286. 10.1007/s00438-022-01856-8 35084582

[B55] Lai Q. Li B. Chen L. Zhou Y. Bao H. Li H. (2025). Substrate stiffness regulates the proliferation and inflammation of chondrocytes and macrophages through exosomes. Acta Biomater. 192, 77–89. 10.1016/j.actbio.2024.12.021 39662715

[B56] Laouteouet D. Bortolotti O. Marineche L. Hannemann N. Chambon M. Glasson Y. (2025). Monocyte-derived macrophages-synovial fibroblasts crosstalk unravels oncostatin signaling network as a driver of synovitis in osteoarthritis. Arthritis Rheumatol. 78, 105–118. 10.1002/art.43299 40621694 PMC12854009

[B57] Lawford B. J. Bennell K. L. Campbell P. K. Kasza J. Hinman R. S. (2021). Association between therapeutic alliance and outcomes following telephone-delivered exercise by a physical therapist for people with knee osteoarthritis: secondary analyses from a randomized controlled trial. JMIR Rehabil. Assist. Technol. 8 (1), e23386. 10.2196/23386 33459601 PMC7850906

[B58] Lawford B. J. Hall M. Hinman R. S. Van der Esch M. Harmer A. R. Spiers L. (2024). Exercise for osteoarthritis of the knee. Cochrane Database Syst. Rev. 12 (12), Cd004376. 10.1002/14651858.CD004376.pub4 39625083 PMC11613324

[B59] Lei Y. Zhan E. Chen C. Hu Y. Lv Z. He Q. (2024). ALKBH5-mediated m(6)A demethylation of Runx2 mRNA promotes extracellular matrix degradation and intervertebral disc degeneration. Cell. Biosci. 14 (1), 79. 10.1186/s13578-024-01264-y 38877576 PMC11179301

[B60] Li H. Yang H. H. Sun Z. G. Tang H. B. Min J. K. (2019). Whole-transcriptome sequencing of knee joint cartilage from osteoarthritis patients. Bone Jt. Res. 8 (7), 290–303. 10.1302/2046-3758.87.Bjr-2018-0297.R1 31463037 PMC6691371

[B61] Li W. Li X. H. Gao Y. Xiong C. J. Tang Z. Z. (2023). Emerging roles of RNA binding proteins in intervertebral disc degeneration and osteoarthritis. Orthop. Surg. 15 (12), 3015–3025. 10.1111/os.13851 37803912 PMC10694020

[B62] Li R. Li D. Xu S. Zhang P. Zhang Z. He F. (2024). Whole-transcriptome sequencing reveals a melanin-related ceRNA regulatory network in the breast muscle of xichuan black-bone chicken. Poult. Sci. 103 (4), 103539. 10.1016/j.psj.2024.103539 38382189 PMC10900940

[B63] Li H. Wang J. Liu Q. Zou Q. Luo X. (2025). Assessment and applications of joint profiling of single-cell chromatin accessibility and transcriptome. Brief. Bioinform 26 (6), bbaf669. 10.1093/bib/bbaf669 41385391 PMC12700103

[B64] Li L. Zhu J. Chen Y. Li H. Han Y. Zhang L. (2025). Interaction between YTH domain-containing family protein 2 and SET domain-containing lysine methyltransferase 7 suppresses autophagy in osteoarthritis chondrocytes, exacerbating cartilage damage. J. Gene Med. 27 (1), e70005. 10.1002/jgm.70005 39789715

[B65] Li R. L. Duan H. X. Liang Q. Huang Y. L. Wang L. Y. Zhang Q. (2022). Targeting matrix metalloproteases: a promising strategy for herbal medicines to treat rheumatoid arthritis. Front. Immunol. 13, 1046810. 10.3389/fimmu.2022.1046810 36439173 PMC9682071

[B66] Li Z. Wang J. Lin Y. Fang J. Xie K. Guan Z. (2022). Newly discovered circRNAs in rheumatoid arthritis, with special emphasis on functional roles in inflammatory immunity. Front. Pharmacol. 13, 983744. 10.3389/fphar.2022.983744 36278188 PMC9585171

[B67] Li Z. Chen Y. Shen Z. (2025). Global shifts in osteoarthritis subtype trends among older adults due to elevated BMI: an age-period-cohort analysis based on the global burden of disease database. Front. Public Health 13, 1518572. 10.3389/fpubh.2025.1518572 40356841 PMC12066270

[B68] Lin X. Tao C. Zhang R. Zhang M. Wang Q. Chen J. (2022). N6-methyladenosine modification of TGM2 mRNA contributes to the inhibitory activity of sarsasapogenin in rheumatoid arthritis fibroblast-like synoviocytes. Phytomedicine 95, 153871. 10.1016/j.phymed.2021.153871 34902811

[B69] Ling H. Wu S. Luo Z. Sun Y. Shen H. Zhou H. (2025). Mechanism by which mechanical stimulation regulates chondrocyte apoptosis and matrix metabolism *via* primary cilia to delay osteoarthritis progression. Zhong Nan Da Xue Xue Bao Yi Xue Ban. 50 (5), 864–875. 10.11817/j.issn.1672-7347.2025.250187 40916823 PMC12406113

[B70] Luo H. Wei J. Wu S. Zheng Q. Zhang N. Chen P. (2023). Exploring CircRNA N6-methyladenosine in human rheumatoid arthritis: hyper-Methylated hsa_circ_0007259 as a potential biomarker and its involvement in the hsa_circ_0007259/hsa_miR-21-5p/STAT3 axis. Int. Immunopharmacol. 124 (Pt A), 110938. 10.1016/j.intimp.2023.110938 37713782

[B71] Lv G. Wang B. Li L. Li Y. Li X. He H. (2022). Exosomes from dysfunctional chondrocytes affect osteoarthritis in sprague-dawley rats through FTO-Dependent regulation of PIK3R5 mRNA stability. Bone Jt. Res. 11 (9), 652–668. 10.1302/2046-3758.119.Bjr-2021-0443.R2 36066338 PMC9533253

[B72] Lv M. Cai Y. Hou W. Peng K. Xu K. Lu C. (2022). The RNA-Binding protein SND1 promotes the degradation of GPX4 by destabilizing the HSPA5 mRNA and suppressing HSPA5 expression, promoting ferroptosis in osteoarthritis chondrocytes. Inflamm. Res. 71 (4), 461–472. 10.1007/s00011-022-01547-5 35320827

[B73] Ma W. Liu Y. Meng C. Luo Y. Wang Q. (2024). Data of RNA-Seq transcriptomes of gastrocnemius muscle, epididymal adipose tissue in Obese rats under normoxia/hypoxic exercise environments. Data Brief. 53, 110134. 10.1016/j.dib.2024.110134 38348322 PMC10859296

[B74] Makaram N. S. Simpson A. (2023). Disease-modifying agents in osteoarthritis: where are we now and what does the future hold? Bone Jt. Res. 12 (10), 654–656. 10.1302/2046-3758.1210.Bjr-2023-0237 37839796 PMC10577043

[B75] Mao G. Xu Y. Long D. Sun H. Li H. Xin R. (2021). Exosome-transported circRNA_0001236 enhances chondrogenesis and suppress cartilage degradation *via* the miR-3677-3p/Sox9 axis. Stem Cell. Res. Ther. 12 (1), 389. 10.1186/s13287-021-02431-5 34256841 PMC8278601

[B76] Mao X. Cao Y. Guo Z. Wang L. Xiang C. (2021). Biological roles and therapeutic potential of circular RNAs in osteoarthritis. Mol. Ther. Nucleic Acids 24, 856–867. 10.1016/j.omtn.2021.04.006 34026329 PMC8131397

[B77] Marushack G. K. Savadipour A. Tang R. Garcia-Castorena J. M. Rashidi N. Nims R. J. (2025). Polyunsaturated fatty acids suppress PIEZO ion channel mechanotransduction in articular chondrocytes. Faseb J. 39 (1), e70290. 10.1096/fj.202400544RR 39786170 PMC13273601

[B78] Nie G. Li Y. Zhao H. Liu C. Zhang Y. Yang X. (2025). Inflammatory microenvironment promotes extracellular matrix degradation of chondrocytes through ALKBH5-dependent Runx2 m(6)A modification in the pathogenesis of osteoarthritis. Int. Immunopharmacol. 144, 113638. 10.1016/j.intimp.2024.113638 39580858

[B79] Nims R. Palmer D. R. Kassab J. Zhang B. Guilak F. (2024). The chondrocyte mechanome: activation of the mechanosensitive ion channels TRPV4 and PIEZO1 drives unique transcriptional signatures. Faseb J. 38 (13), e23778. 10.1096/fj.202400883R 38959010 PMC11327906

[B80] Oo W. M. (2024). Prospects of disease-modifying osteoarthritis drugs. Rheum. Dis. Clin. North Am. 50 (3), 483–518. 10.1016/j.rdc.2024.03.003 38942581

[B81] Pan X. Zhao Z. Huang X. Cen X. (2023). Circ-Slain2 alleviates cartilage degradation and inflammation of TMJOA. J. Dent. Res. 102 (13), 1498–1506. 10.1177/00220345231198448 37817544

[B82] Pan Y. Zhang F. Zhong J. Gong Q. Geng N. Kuang B. (2026). CircTspan3 promotes cartilage development through ANNEXIN A2-Mediated ferroptosis and apoptosis inhibition and exosome-mediated paracrine signaling. Adv. Sci. (Weinh) 13, e13418. 10.1002/advs.202513418 41486887 PMC12915206

[B83] Peitola J. P. J. Esrafilian A. Simonsen M. B. Andersen M. S. Korhonen R. K. (2025). Reduced muscle strength can alter the impact of gait modifications on knee cartilage mechanics. J. Orthop. Res. 43 (9), 1566–1580. 10.1002/jor.70007 40576011 PMC12329646

[B84] Pérez-García S. Gutiérrez-Cañas I. Seoane I. V. Fernández J. Mellado M. Leceta J. (2016). Healthy and osteoarthritic synovial fibroblasts produce a disintegrin and metalloproteinase with thrombospondin motifs 4, 5, 7, and 12: induction by IL-1β and fibronectin and contribution to cartilage damage. Am. J. Pathol. 186 (9), 2449–2461. 10.1016/j.ajpath.2016.05.017 27449198

[B85] Pérez-García S. Carrión M. Gutiérrez-Cañas I. Villanueva-Romero R. Castro D. Martínez C. (2019). Profile of matrix-remodeling proteinases in osteoarthritis: impact of fibronectin. Cells 9 (1), 40. 10.3390/cells9010040 31877874 PMC7017325

[B86] Qin C. Feng Y. Yin Z. Wang C. Yin R. Li Y. (2024). The PIEZO1/miR-155-5p/GDF6/SMAD2/3 signaling axis is involved in inducing the occurrence and progression of osteoarthritis under excessive mechanical stress. Cell. Signal 118, 111142. 10.1016/j.cellsig.2024.111142 38508350

[B87] Que W. Liu H. Yang Q. (2022). CircPRKCH modulates extracellular matrix formation and metabolism by regulating the miR-145/HGF axis in osteoarthritis. Arthritis Res. Ther. 24 (1), 216. 10.1186/s13075-022-02893-9 36068644 PMC9447342

[B88] Quero L. Tiaden A. N. Hanser E. Roux J. Laski A. Hall J. (2019). miR-221-3p drives the shift of M2-Macrophages to a pro-inflammatory function by suppressing JAK3/STAT3 activation. Front. Immunol. 10, 3087. 10.3389/fimmu.2019.03087 32047494 PMC6996464

[B89] Ren S. Lin P. Wang J. Yu H. Lv T. Sun L. (2020). Circular RNAs: promising molecular biomarkers of human aging-related diseases *via* functioning as an miRNA sponge. Mol. Ther. Methods Clin. Dev. 18, 215–229. 10.1016/j.omtm.2020.05.027 32637451 PMC7326721

[B90] Ren X. Zhuang H. Li B. Jiang F. Zhang Y. Zhou P. (2023). Gsmtx4 alleviated osteoarthritis through Piezo1/Calcineurin/NFAT1 signaling axis under excessive mechanical strain. Int. J. Mol. Sci. 24 (4), 4022. 10.3390/ijms24044022 36835440 PMC9961447

[B91] Rodriguez-Merchan E. C. (2023). The current role of disease-modifying osteoarthritis drugs. Arch. Bone Jt. Surg. 11 (1), 11–22. 10.22038/abjs.2021.56530.2807 36793668 PMC9903308

[B92] Sacco I. C. N. Trombini-Souza F. Suda E. Y. (2023). Impact of biomechanics on therapeutic interventions and rehabilitation for major chronic musculoskeletal conditions: a 50-year perspective. J. Biomech. 154, 111604. 10.1016/j.jbiomech.2023.111604 37159980

[B93] Shang X. Böker K. O. Taheri S. Lehmann W. Schilling A. F. (2021). Extracellular vesicles allow epigenetic mechanotransduction between chondrocytes and osteoblasts. Int. J. Mol. Sci. 22 (24), 13282. 10.3390/ijms222413282 34948080 PMC8703680

[B94] Shao Z. Tu Z. Shi Y. Li S. Wu A. Wu Y. (2020). RNA-binding protein HuR suppresses inflammation and promotes extracellular matrix homeostasis *via* NKRF in intervertebral disc degeneration. Front. Cell. Dev. Biol. 8, 611234. 10.3389/fcell.2020.611234 33330514 PMC7732619

[B95] Shao Y. Zhang H. Guan H. Wu C. Qi W. Yang L. (2024). PDZK1 protects against mechanical overload-induced chondrocyte senescence and osteoarthritis by targeting mitochondrial function. Bone Res. 12 (1), 41. 10.1038/s41413-024-00344-6 39019845 PMC11255281

[B96] Shen P. Yang Y. Liu G. Chen W. Chen J. Wang Q. (2020). CircCDK14 protects against osteoarthritis by sponging miR-125a-5p and promoting the expression of Smad2. Theranostics 10 (20), 9113–9131. 10.7150/thno.45993 32802182 PMC7415803

[B97] Shen S. Yang Y. Shen P. Ma J. Fang B. Wang Q. (2021). circPDE4B prevents articular cartilage degeneration and promotes repair by acting as a scaffold for RIC8A and MID1. Ann. Rheum. Dis. 80 (9), 1209–1219. 10.1136/annrheumdis-2021-219969 34039624 PMC8372377

[B98] Shen P. Gao J. Huang S. You C. Wang H. Chen P. (2023). LncRNA AC006064.4-201 serves as a novel molecular marker in alleviating cartilage senescence and protecting against osteoarthritis by destabilizing CDKN1B mRNA *via* interacting with PTBP1. Biomark. Res. 11 (1), 39. 10.1186/s40364-023-00477-6 37055817 PMC10099822

[B99] Shi S. Zhao R. (2025). RNA-binding proteins (RBPs) and circular RNA biogenesis. Adv. Exp. Med. Biol. 1485, 117–130. 10.1007/978-981-96-9428-0_8 40886273 PMC13105153

[B100] Singh M. Roshan K. Muthuswamy S. Kumar A. Kumar S. (2025). Circular RNAs binding to RNA-binding proteins. Adv. Exp. Med. Biol. 1485, 151–167. 10.1007/978-981-96-9428-0_10 40886275

[B101] Steinecker-Frohnwieser B. Lohberger B. Toegel S. Windhager R. Glanz V. Kratschmann C. (2023). Activation of the mechanosensitive ion channels Piezo1 and TRPV4 in primary human healthy and osteoarthritic chondrocytes exhibits ion channel crosstalk and modulates gene expression. Int. J. Mol. Sci. 24 (9), 7868. 10.3390/ijms24097868 37175575 PMC10178441

[B102] Su J. Chen S. Yang S. Deng Z. (2024). RNA-Binding proteins regulate osteoarthritis *via* RNA metabolism regulation. Zhong Nan Da Xue Xue Bao Yi Xue Ban. 49 (12), 1973–1982. 10.11817/j.issn.1672-7347.2024.240261 40195670 PMC11975523

[B103] Sun K. Lu F. Hou L. Zhang X. Pan C. Liu H. (2024). IRF1 regulation of ZBP1 links mitochondrial DNA and chondrocyte damage in osteoarthritis. Cell. Commun. Signal 22 (1), 366. 10.1186/s12964-024-01744-1 39026271 PMC11256489

[B104] Sun H. Sun L. Liu M. Zhuang Y. Ning X. Yang H. (2025). Global burden of high BMI-Related osteoarthritis and causal effects of obesity: a GBD 1990-2021 and bidirectional two-sample Mendelian randomization study. Int. J. Surg. 111 (12), 9110–9120. 10.1097/js9.0000000000003133 40788017 PMC12695367

[B105] Sun W. Liao Y. Feng J. Liang J. He Q. Cui Y. (2026). Targeting of CIRP attenuates osteoarthritis progression *via* suppressing TLR4/NF-κB/NLRP3 signaling axis. Int. J. Mol. Med. 57 (1), 3. 10.3892/ijmm.2025.5674 41133454 PMC12582850

[B106] Suo J. Li L. Tan W. Yin X. Wang J. Shao R. (2025). Circular RNA-Based protein replacement therapy mitigates osteoarthritis in Male mice. Nat. Commun. 16 (1), 8480. 10.1038/s41467-025-63343-z 41006212 PMC12474924

[B107] Swahn H. Olmer M. Lotz M. K. (2023). RNA-Binding proteins that are highly expressed and enriched in healthy cartilage but suppressed in osteoarthritis. Front. Cell. Dev. Biol. 11, 1208315. 10.3389/fcell.2023.1208315 37457300 PMC10349536

[B108] Tang Y. Zhang D. Luo H. (2025). YTHDF3 stabilizes the expression of m6A-Mediated LRRC17 and induces osteoarthritic chondrocyte senescence by activating the STAT1 signaling. Geriatr. Gerontol. Int. 26, e70269. 10.1111/ggi.70269 41365291

[B109] Tokura T. Matsushita T. Nishida K. Nagai K. Kanzaki N. Hoshino Y. (2025). SIRT1 antisense long noncoding RNA attenuates interleukin-1β-induced osteoarthritic gene expression in human chondrocytes through its mRNA interaction. Sci. Rep. 15 (1), 23338. 10.1038/s41598-025-06565-x 40603391 PMC12223068

[B110] Wang L. Ye Y. (2024). Trends and projections of the burden of osteoarthritis disease in China and globally: a comparative study of the 2019 global burden of disease database. Prev. Med. Rep. 37, 102562. 10.1016/j.pmedr.2023.102562 38205169 PMC10776652

[B111] Wang B. Y. Chen Y. F. Hsiao A. W. Chen W. J. Lee C. W. Lee O. K. (2023). Ginkgolide B facilitates muscle regeneration *via* rejuvenating osteocalcin-mediated bone-to-muscle modulation in aged mice. J. Cachexia Sarcopenia Muscle 14 (3), 1349–1364. 10.1002/jcsm.13228 37076950 PMC10235878

[B112] Wang D. Zhang Z. Li X. He L. (2024). RNA binding protein PUM2 promotes IL-1β-induced apoptosis of chondrocytes *via* regulating FOXO3 expression. Heliyon 10 (3), e25080. 10.1016/j.heliyon.2024.e25080 38356524 PMC10865267

[B113] Wang X. Ji Y. Feng P. Liu R. Li G. Zheng J. (2021). The m6A reader IGF2BP2 regulates macrophage phenotypic activation and inflammatory diseases by stabilizing TSC1 and PPARγ. Adv. Sci. (Weinh) 8 (13), 2100209. 10.1002/advs.202100209 34258163 PMC8261491

[B114] Wang Y. Ma X. Chen Y. Xia C. Lei X. Liu J. (2025). Slo1 deficient myoblast exosomes-derived miR-222-3p inhibits osteogenic differentiation *via* targeting of STAT3. J. Cachexia Sarcopenia Muscle 16 (6), e70115. 10.1002/jcsm.70115 41360438 PMC12685407

[B115] Wang H. Su J. Yu M. Xia Y. Wei Y. (2023). PGC-1α in osteoarthritic chondrocytes: from mechanism to target of action. Front. Pharmacol. 14, 1169019. 10.3389/fphar.2023.1169019 37089944 PMC10117990

[B116] Wang H. Zhang Y. Zhang C. Zhao Y. Shu J. Tang X. (2024). Exosomes derived from miR-146a-overexpressing fibroblast-like synoviocytes in cartilage degradation and macrophage M1 polarization: a novel protective agent for osteoarthritis? Front. Immunol. 15, 1361606. 10.3389/fimmu.2024.1361606 38846937 PMC11153682

[B117] Wang S. Li W. Zhang P. Wang Z. Ma X. Liu C. (2022). Mechanical overloading induces GPX4-regulated chondrocyte ferroptosis in osteoarthritis *via* Piezo1 channel facilitated calcium influx. J. Adv. Res. 41, 63–75. 10.1016/j.jare.2022.01.004 36328754 PMC9637484

[B118] Wang Z. Rao Z. Wang X. Jiang C. Yang Y. (2022). circPhc3 sponging microRNA-93-3p is involved in the regulation of chondrocyte function by mechanical instability in osteoarthritis. Int. J. Mol. Med. 49 (1), 6. 10.3892/ijmm.2021.5061 34779488 PMC8612303

[B119] Wood M. K. Daoud A. Talor M. V. Kalinoski H. M. Hughes D. M. Jaime C. M. (2024). Programmed death ligand 1-Expressing macrophages and their protective role in the joint during arthritis. Arthritis Rheumatol. 76 (4), 553–565. 10.1002/art.42749 37997621 PMC12506893

[B120] Wu Y. Hong Z. Xu W. Chen J. Wang Q. Chen J. (2021). Circular RNA circPDE4D protects against osteoarthritis by binding to miR-103a-3p and regulating FGF18. Mol. Ther. 29 (1), 308–323. 10.1016/j.ymthe.2020.09.002 33125858 PMC7791010

[B121] Wu Y. Hou M. Deng Y. Xia X. Liu Y. Yu J. (2025). Swimming exercise induces redox-lipid crosstalk to ameliorate osteoarthritis progression. Redox Biol. 81, 103535. 10.1016/j.redox.2025.103535 39952199 PMC11875157

[B122] Xiang Q. Kang L. Wang J. Liao Z. Song Y. Zhao K. (2020). CircRNA-CIDN mitigated compression loading-induced damage in human nucleus pulposus cells *via* miR-34a-5p/SIRT1 axis. EBioMedicine 53, 102679. 10.1016/j.ebiom.2020.102679 32114390 PMC7044714

[B123] Xiang M. Liu L. Wu T. Wei B. Liu H. (2023). RNA-Binding proteins in degenerative joint diseases: a systematic review. Ageing Res. Rev. 86, 101870. 10.1016/j.arr.2023.101870 36746279

[B124] Xie X. Zhang K. Li Y. Li Y. Li X. Lin Y. (2025). Global, regional, and national burden of osteoarthritis from 1990 to 2021 and projections to 2035: a cross-sectional study for the global burden of disease study 2021. PLoS One 20 (5), e0324296. 10.1371/journal.pone.0324296 40424273 PMC12111611

[B125] Xu S. Liu D. Kuang Y. Li R. Wang J. Shi M. (2023). Long noncoding RNA HAFML promotes migration and invasion of rheumatoid fibroblast-like synoviocytes. J. Immunol. 210 (2), 135–147. 10.4049/jimmunol.2200453 36458981

[B126] Xu Y. Liu W. Ren L. (2024). Emerging roles and mechanism of m6A methylation in rheumatoid arthritis. Biomed. Pharmacother. 170, 116066. 10.1016/j.biopha.2023.116066 38157641

[B127] Xu Y. Yang Y. Song H. Li M. Shi W. Yu T. (2025). The role of exerkines in the treatment of knee osteoarthritis: from mechanisms to exercise strategies. Orthop. Surg. 17 (4), 1021–1035. 10.1111/os.14365 39854050 PMC11962297

[B128] Yang D. Xu J. Xu K. Xu P. (2024). Skeletal interoception in osteoarthritis. Bone Res. 12 (1), 22. 10.1038/s41413-024-00328-6 38561376 PMC10985098

[B129] Yang D. Yang L. Yang W. Chen J. Wang S. Yang J. (2025). Mechano-metabolic dysregulation in osteoarthritis: piezo1-Mediated mitophagy impairment as a novel therapeutic target. Inflamm. Res. 75 (1), 6. 10.1007/s00011-025-02161-x 41410756

[B130] Yao N. Peng S. Wu H. Liu W. Cai D. Huang D. (2022). Long noncoding RNA PVT1 promotes chondrocyte extracellular matrix degradation by acting as a sponge for miR-140 in IL-1β-stimulated chondrocytes. J. Orthop. Surg. Res. 17 (1), 218. 10.1186/s13018-022-03114-4 35399100 PMC8996637

[B131] Yi Q. Deng Z. Yue J. He J. Xiong J. Sun W. (2022). RNA binding proteins in osteoarthritis. Front. Cell. Dev. Biol. 10, 954376. 10.3389/fcell.2022.954376 36003144 PMC9393224

[B132] Yoon D. S. Lee K. M. Choi Y. Ko E. A. Lee N. H. Cho S. (2022). TLR4 downregulation by the RNA-Binding protein PUM1 alleviates cellular aging and osteoarthritis. Cell. Death Differ. 29 (7), 1364–1378. 10.1038/s41418-021-00925-6 35034101 PMC9287402

[B133] Yu Y. Su Y. Wang G. Lan M. Liu J. Garcia Martin R. (2024). Reciprocal communication between FAPs and muscle cells *via* distinct extracellular vesicle miRNAs in muscle regeneration. Proc. Natl. Acad. Sci. U. S. A. 121 (11), e2316544121. 10.1073/pnas.2316544121 38442155 PMC10945765

[B134] Yu Z. Li X. Huang J. Pan J. Cheng J. Liu P. (2025). The RNA-Binding E3 ligase MKRN2 selectively disrupts Il6 translation to restrain inflammation. Nat. Immunol. 26 (7), 1036–1047. 10.1038/s41590-025-02183-x 40524017

[B135] Zeng T. Liu W. Q. Zhou Y. H. Ye S. F. Chen Z. L. (2025). Osteoarthritis burden across 204 countries and territories during 1990 to 2021: insights from the global burden of disease study 2021. Med. Baltim. 104 (51), e46612. 10.1097/MD.0000000000046612 41431043 PMC12727419

[B136] Zhang B. Chen Y. Chen Q. Zhang H. (2025). Exosomal miRNAs in muscle-bone crosstalk: mechanistic links, exercise modulation and implications for sarcopenia, osteoporosis and osteosarcopenia. Metabolism 170, 156333. 10.1016/j.metabol.2025.156333 40550325

[B137] Zhang C. Chen Y. Zhang J. (2025). Non-coding RNAs in osteoarthritis and osteoporosis-related hip fractures: molecular biomarkers for precision diagnosis and prognosis. Front. Endocrinol. 16, 1653831. 10.3389/fendo.2025.1653831 41158629 PMC12554559

[B138] Zhang H. Cai D. Bai X. (2020). Macrophages regulate the progression of osteoarthritis. Osteoarthr. Cartil. 28 (5), 555–561. 10.1016/j.joca.2020.01.007 31982565

[B139] Zhang F. Jonsson A. H. Nathan A. Millard N. Curtis M. Xiao Q. (2023). Deconstruction of rheumatoid arthritis synovium defines inflammatory subtypes. Nature 623 (7987), 616–624. 10.1038/s41586-023-06708-y 37938773 PMC10651487

[B140] Zhang H. Shao Y. Yao Z. Liu L. Zhang H. Yin J. (2022). Mechanical overloading promotes chondrocyte senescence and osteoarthritis development through downregulating FBXW7. Ann. Rheum. Dis. 81 (5), 676–686. 10.1136/annrheumdis-2021-221513 35058228

[B141] Zhang K. Wang Z. He J. Lu L. Wang W. Yang A. (2025). Mechanisms of synovial macrophage polarization in osteoarthritis pathogenesis and their therapeutic implications. Front. Immunol. 16, 1637731. 10.3389/fimmu.2025.1637731 41376619 PMC12685870

[B142] Zhang M. Xiong W. Qiao R. Li M. Zhang C. Yang C. (2025). Irisin in the modulation of bone and cartilage homeostasis: a review on osteoarthritis relief potential. Front. Physiol. 16, 1570157. 10.3389/fphys.2025.1570157 40313878 PMC12043700

[B143] Zhang P. Wu W. Ma C. Du C. Huang Y. Xu H. (2022). RNA-binding proteins in the regulation of adipogenesis and adipose function. Cells 11 (15), 2357. 10.3390/cells11152357 35954201 PMC9367552

[B144] Zhang S. Zhang B. Liao Z. Chen Y. Guo W. Wu J. (2024). Hnrnpk protects against osteoarthritis through targeting WWC1 mRNA and inhibiting hippo signaling pathway. Mol. Ther. 32 (5), 1461–1478. 10.1016/j.ymthe.2024.02.027 38414246 PMC11081807

[B145] Zhang Y. Liu L. Liu K. Wang M. Su X. Wang J. (2022). Regulatory mechanism of circular RNA involvement in osteoarthritis. Front. Surg. 9, 1049513. 10.3389/fsurg.2022.1049513 36684373 PMC9852714

[B146] Zhang Z. Dong L. Tao H. Dong Y. Xiang W. Tao F. (2024). RNA-Binding proteins potentially regulate the alternative splicing of apoptotic genes during knee osteoarthritis progression. BMC Genomics 25 (1), 293. 10.1186/s12864-024-10181-w 38504181 PMC10949708

[B147] Zhou G. Zhang X. Gu Z. Zhao J. Luo M. (2024). Research progress in single-herb Chinese medicine and compound medicine for knee osteoarthritis. Comb. Chem. High. Throughput Screen 27 (15), 2180–2186. 10.2174/0113862073264850231116055745 38305402 PMC11348453

[B148] Zhou J. Zhang Y. Xie S. Li Y. Sun Q. Tang X. (2025). The escalating burden of osteoarthritis in east and Southeast Asia: an information-analytical study from the GBD 2021. Clin. Rheumatol. 44 (12), 4819–4832. 10.1007/s10067-025-07740-1 41094301 PMC12602588

[B149] Zhou R. Li K. Zhang H. Wang Y. Wei C. Fan S. (2026). Urolithin A attenuates sleep deprivation-induced neutrophilic inflammation by suppression of the ROS-H3K18la feedback loop. J. Agric. Food Chem. 74, 2773–2788. 10.1021/acs.jafc.5c10266 41524914

[B150] Zhou Y. Li M. Jin K. Wen M. Qin H. Xu Y. (2025). The RNA-Binding protein RRP1 brakes macrophage one-carbon metabolism to suppress autoinflammation. Nat. Commun. 16 (1), 6880. 10.1038/s41467-025-62173-3 40715096 PMC12297237

[B151] Zhou Z. Li Y. Xi K. Wu Y. Jia Y. Si Y. (2026). Gubi zhitong formula alleviates knee osteoarthritis induced by excessive mechanical loading in rats *via* regulating Piezo1 mediated Ca(2+) signaling pathway and phosphoinositide 3-Kinase/protein kinase B/nuclear factor kappa-B axis. J. Ethnopharmacol. 356, 120831. 10.1016/j.jep.2025.120831 41173085

[B152] Zhu Z. Xie J. Manandhar U. Yao X. Bian Y. Zhang B. (2021). RNA binding protein GNL3 up-regulates IL24 and PTN to promote the development of osteoarthritis. Life Sci. 267, 118926. 10.1016/j.lfs.2020.118926 33358901

[B153] Zhu Q. Yin F. Qin J. Shi W. Liu Y. Zhao Y. (2025). Procr(+) chondroprogenitors sense mechanical stimuli to govern articular cartilage maintenance and regeneration. Cell. 188 (19), 5194–5211.e5116. 10.1016/j.cell.2025.06.036 40695281

[B154] Zigdon I. Carmi M. Brodsky S. Rosenwaser Z. Barkai N. Jonas F. (2024). Beyond RNA-Binding domains: determinants of protein-RNA binding. Rna 30 (12), 1620–1633. 10.1261/rna.080026.124 39353735 PMC11571813

